# Accelerating lithium-ion pre-desolvation and transport via glassy MOF for fast-charging and high-energy-density lithium-ion batteries

**DOI:** 10.1093/nsr/nwaf349

**Published:** 2025-08-22

**Authors:** Yan Xu, Danni Zhang, Shibin Zhang, Lishun Bai, Yue Liu, Jingwen Zhao, Zhi Chang, Haoshen Zhou

**Affiliations:** School of Materials Science and Engineering, Key Laboratory of Electronic Packaging and Advanced Functional Materials of Hunan Province, Central South University, Changsha 410083, China; School of Materials Science and Engineering, Key Laboratory of Electronic Packaging and Advanced Functional Materials of Hunan Province, Central South University, Changsha 410083, China; School of Materials Science and Engineering, Key Laboratory of Electronic Packaging and Advanced Functional Materials of Hunan Province, Central South University, Changsha 410083, China; School of Materials Science and Engineering, Key Laboratory of Electronic Packaging and Advanced Functional Materials of Hunan Province, Central South University, Changsha 410083, China; School of Materials Science and Engineering, Key Laboratory of Electronic Packaging and Advanced Functional Materials of Hunan Province, Central South University, Changsha 410083, China; Center of Energy Storage Materials & Technology, College of Engineering and Applied Sciences, Jiangsu Key Laboratory of Artificial Functional Materials, National Laboratory of Solid State Micro-structures, and Collaborative Innovation Center of Advanced Micro-structures, Nanjing University, Nanjing 210093, China; School of Materials Science and Engineering, Key Laboratory of Electronic Packaging and Advanced Functional Materials of Hunan Province, Central South University, Changsha 410083, China; Center of Energy Storage Materials & Technology, College of Engineering and Applied Sciences, Jiangsu Key Laboratory of Artificial Functional Materials, National Laboratory of Solid State Micro-structures, and Collaborative Innovation Center of Advanced Micro-structures, Nanjing University, Nanjing 210093, China

**Keywords:** lithium-ion battery, glassy metal-organic framework, pre-desolvation, fast-charging, high energy density

## Abstract

Conventional graphite anodes in lithium-ion batteries (LIBs) suffer from limited fast-charging capability and lithium dendrite growth, particularly at high current densities. This work introduces a glassy metal-organic framework (MOF glass) that simultaneously enables easy lithium-ion pre-desolvation and fast Li⁺ transport. The MOF glass-coated graphite (glass@graphite) forms a distinctive double-layer structure during initial discharge: an electron-insulating outer layer with rigid 2.93 Å pores that facilitates easy Li⁺ pre-desolvation, and a Li₃P-rich inner layer that ensures rapid lithium-ion conduction. The outer layer's pre-desolvation effect generates a highly aggregated electrolyte within MOF channels, promoting formation of a stable anion-derived LiF-dominated solid electrolyte interphase. The resulting partially desolvated Li⁺ species readily penetrate the ion-conducting inner layer, enabling ultrafast diffusion. When coupled with LiNi_0.8_Co_0.1_Mn_0.1_O_2_ (NCM-811) cathodes, the NCM-811//glass@graphite full cells demonstrate remarkable fast-charging performance (88% capacity retention after 1000 cycles at a high current of 4 C). A practical 2.36 Ah pouch cell achieves an energy density of 283 Wh/kg while maintaining over 80% capacity after 300 cycles. This approach presents a transformative strategy for developing fast-charging, high-energy-density LIBs.

## Teaser

Glassy MOFs overcomes graphiteas slow desolvation bottleneck in lithium-ion batteries, paving the way for electric vehicle batteries that charge in minutes and last much longer per charge.

## INTRODUCTION

**Figure 1.**
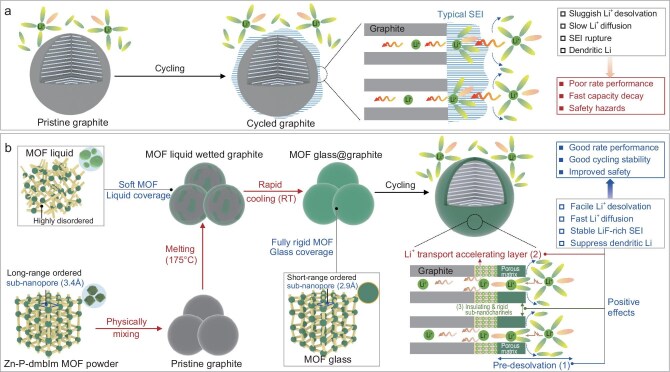
The positive effects of coating graphite with glassy MOF facilitate lithium-ion pre-desolvation and accelerate fast lithium-ion transport. (a) Schematic illustration of the challenges and negative effects commercial graphite experiences during electrochemical cycling. (b) Schematic illustration of the preparation process and the benefits of coating commercial graphite with 2.93 Å MOF glass containing P elements. During cycling, the single MOF glass layer is transformed into a double-layer structure. The outer layer, made of electron-insulating MOF glass with rigid 2.93 Å pores, promotes efficient lithium-ion pre-desolvation and facilitates the formation of a highly concentrated electrolyte with compact partially desolvated lithium ions within the MOF glass channels. The inner section, in direct contact with graphite, forms a lithium-ion accelerating layer that enhances rapid lithium-ion transport.

With the rapid development of technology in society, rechargeable batteries, especially lithium-ion batteries (LIBs), are widely used in the fields of electric vehicles (EVs) and electronic devices due to their high energy density and long cycle life [1–4]. Graphite, with its low lithium-ion intercalation potential (∼0.1 V vs Li/Li^+^) and high theoretical capacity (372 mAh/g), has become a dominant anode material in LIBs [5,6]. However, the uneven solid–electrolyte interphase (SEI) formed on the graphite surface during the charge–discharge process, especially under fast charging, significantly hinders the lithium-ion desolvation and slows down the diffusion of lithium ions, resulting in dissatisfactory rate performance [7–9]. Even worse, the continuous collapse and regeneration of the SEI layer depletes electrolyte and causes dendrite formation, leading to poor rate performance, fast capacity fading and, ultimately, thermal runaway and cell deformation [10–12] (as schematically illustrated in Fig. 1a).

Currently, high-concentration electrolytes (HCEs) [12–16] and coating modifications [17–20] offer promising solutions to mitigate these issues. In HCEs, anions are preferentially reduced, forming a stable inorganic-rich SEI layer that reduces the accompanying side reactions with free solvent molecules and the lithium dendrites in typical electrolytes [21,22], thus improving cycle stability of the batteries. However, increasing salt concentrations generally leads to higher electrolyte viscosity [23,24], which would slow lithium-ion transport and degrade interface performance, hindering the rate capability. Furthermore, the high cost of HCEs greatly limits their further commercialization. Coating strategies indeed improve the specific capacity and mechanical stability of graphite by providing additional Li^+^ ion storage sites and prevent direct contact between graphite and electrolyte, reducing byproducts [19,25]. However, conventional coating layers tend to hinder lithium-ion migration and diffusion [26,27], making fast-charging LIBs highly challenging. Therefore, more effective solutions are urgently needed to further improve the electrochemical performance of LIBs.

Recent studies have shown that coating electrodes with electro-insulating, porous materials such as metal–organic frameworks (MOFs) [28–32], zeolites [33] and covalent organic frameworks (COFs) [34–36] can promote lithium-ion pre-desolvation and effectively suppress solvent-related decomposition, thereby enhancing battery electrochemical performance. However, this lithium-ion pre-desolvation strategy involves directly coating electrodes (electrode-level coating) with porous materials mixed with polymer binders, which inevitably introduces cracks and gaps [37,38]. These defects, often ranging from micrometers to tens of micrometers in thickness, counteract the benefits of pre-desolvation and hinder fast lithium-ion diffusion. Therefore, achieving particle-level ultra-thin coatings that enable complete lithium-ion pre-desolvation is highly desirable. Ideally, a lithium-ion accelerating layer formed between the coating and graphite would further enhance fast-charging performance. However, conventional methods struggle to achieve both effects simultaneously, hindering the development of high-energy-density, fast-charging LIBs.

In this work, we found that by coating graphite with 2.93 Å MOF powder (Zn–P–dmbIm, where P = phosphate, dmbIm = 5,6-dimethylbenzimidazole), followed by heat treatment at 175°C, a glassy MOF-coated graphite (glass@graphite) with an ultra-thin 5 nm thickness was obtained [39]. After the first discharge cycle, the single-layered glass@graphite composite transformed into a special double-layered structure. Specifically, the outer layer was the rigid, electron-insulating MOF glass with narrow 2.93 Å pore windows, promoting efficient lithium-ion pre-desolvation and forming a highly concentrated electrolyte of partially desolvated, smaller lithium ions within the MOF channels. These partially desolvated lithium ions enabled the anion-derived LiF-dominated SEI layer and suppressed dendritic lithium formation. The inner layer, in direct contact with graphite, formed Li_3_P, enhancing the transport of partially desolvated, smaller lithium ions and enabling faster charging (as schematically illustrated in Fig. 1b). Benefits from the unique double-layered structure, the glass@graphite anode delivered an ultra-high capacity of more than 250 mAh/g at a high current density of 5 C, five times higher than that of pristine graphite. The cycled glass@graphite anode maintained a dendrite-free structure with no obvious byproduct formation, even under such harsh fast-charging conditions. Moreover, after coupling the glass@graphite with the LiNi_0.8_Co_0.1_Mn_0.1_O_2_ (NCM-811) cathode, the NCM-811//glass@graphite full cell acquired excellent rate performance and long-term cycle stability (preserved 88% capacity retention after 1000 cycles at 4 C current density). Notably, a 2.36 Ah NCM-811//glass@graphite pouch cell achieved an impressive energy density of 283 Wh/kg and retained 80% of its initial capacity after 300 cycles, highlighting its promise for high-energy-density battery advancements.

## RESULTS AND DISCUSSION

### Preparation and characterizations of MOF glass-coated graphite (glass@graphite) anode

**Figure 2.**
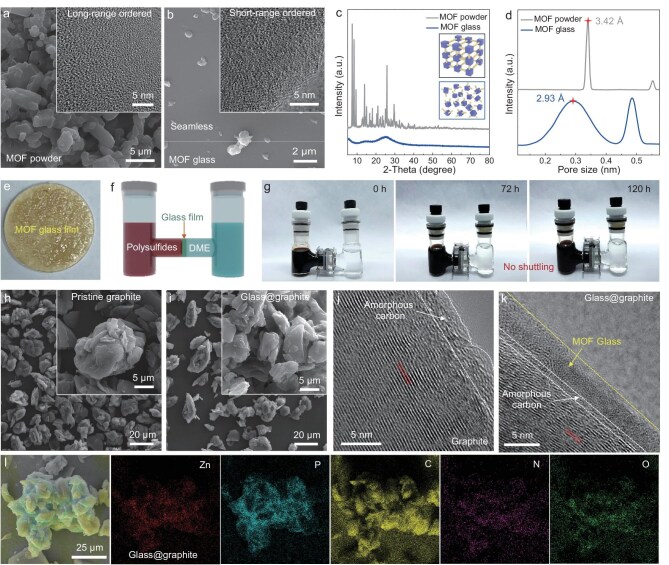
Characterizations of the MOF glass-coated graphite (glass@graphite). Scanning electron microscopy (SEM) and transmission electron microscopy (TEM) images of (a) Zn–P–dmbIm MOF powder and (b) MOF glass. (c) XRD patterns of Zn–P–dmbIm MOF powder and MOF glass. (d) Pore size distribution of the Zn–P–dmbIm MOF powder and MOF glass. (e–g) Polysulfide permeation test used to verify the crack-free property of the MOF glass film. SEM and high-resolution TEM (HRTEM) images of (h and j) pristine graphite and (i and k) glass@graphite coated with uniform and ultra-thin MOF glass featuring subnanochannels. (l) Energy-dispersive spectroscopy (EDS) elemental mappings of Zn, P, C, N and O for glass@graphite.

Critically, conventional porous coating materials are typically applied directly onto electrode surfaces, confining lithium-ion pre-desolvation only to the electrode level, while achieving uniform particle-level pre-desolvation remains challenging due to their particulate powdery morphology. This inherent discontinuity prevents comprehensive particle coverage. More importantly, effective acceleration of lithium-ion diffusion requires phosphorus (P) incorporation—which forms Li_3_P upon lithiation to enhance ion transport kinetics [40–43]. With these factors in mind, we selected a unique MOF, Zn–P–dmbIm (Figs S1 and S2a), to fulfill our goal. The Zn–P–dmbIm MOF possessed 3.42 Å pore windows [39], much smaller than lithium ions, which can promote the pre-desolvation of lithium ions [37,38,44]. Moreover, the P element within Zn–P–dmbIm MOF was expected to form Li_3_P, which consequently promoted fast lithium-ion diffusion. Most importantly, the Zn–P–dmbIm MOF can transition into a flowing liquid (MOF liquid) before vitrifying into a glassy state (MOF glass possessed even much narrower pore windows of about 2.93 Å). The Zn–P–dmbIm MOF powder changed from solid to liquid upon heating to its melting temperature (*T*_m_). Cooling the molten MOF liquid from a temperature slightly above *T*_m_ to room temperature yielded MOF glass. Thermogravimetric analysis (TGA) and differential scanning calorimetry (DSC) measurements were performed in an inert atmosphere to determine the precise thermal responses of samples. The Zn–P–dmbIm MOF powder demonstrated a clear endothermic peak at about 173.5°C (*T*_m_) before decomposition (decomposition temperature, *T*_d_ = 218.2°C) (Fig. S3a), and the subsequently cyclic DSC scans of the prepared glass indicated that the glass transition temperature (*T*_g_) was about 111.7°C (Fig. S3b). Based on the TGA-DSC result, we successfully prepared the MOF glass. Obviously, the Zn–P–dmbIm MOF powder (hereafter MOF powder) and the MOF glass demonstrated totally different morphologies. The former was composed of various particles, while the latter demonstrated a crack-free, film-like structure (Fig. 2a and b). Notably, unlike the MOF powder, which exhibited a distinct long-range ordered structure, the MOF glass displayed a short-range ordered structure (inset in Fig. 2a and b, Figs S4–S9). In addition, those two different MOF states also led to distinct X-ray diffraction (XRD) patterns, in which the MOF powder exhibited sharp crystalline peaks while the MOF glass did not show any obvious crystalline peaks (Fig. 2c). This further confirmed the successful transformation of crystalline MOF powder to amorphous MOF glass. The corresponding attenuated total reflection Fourier transform infrared (ATR-FTIR) spectra exhibited negligible variation before and after vitrification (Fig. S10), confirming retention of a nearly identical coordination environment in the glass phase relative to its crystalline precursor, with no new chemical bonds generated. The pore size distributions of MOF powder and MOF glass derived from positron annihilation lifetime spectroscopy (PALS) measurements were expressed as a Gaussian distribution. Clearly, after vitrification, the pore size of MOF glass was only 2.93 Å, much smaller than that of its counterpart, which was 3.42 Å (Fig. 2d and Table S1). Notably, the prepared MOF glass film was crack-free and effectively suppressed the crossover of small polysulfides (∼9.0 Å) for 120 h (Fig. 2e–g). Based on these unique properties of MOF glass, we can coat the graphite particles with MOF liquid completely and evenly (Fig. S2b, defined as glass@graphite). In order to explore the best conditions for glass coating, glass@graphite with different amounts of MOF glass coating were successfully prepared (proportions of MOF glass were 2 wt%, 5 wt% and 10 wt% and correspondingly denoted as 2 wt% glass@graphite, 5 wt% glass@graphite and 10 wt% glass@graphite, Figs S11–S18). Clearly, 2 wt% MOF glass was sufficient to fully coat the surface of graphite particles. Further increasing the MOF glass content only resulted in a thicker coating layer. Therefore, the glass@graphite sample with a 2 wt% MOF glass coating was chosen as the main sample (Fig. 2i and k, Figs S11–S18). The overall morphology of the glass@graphite particles was basically unchanged after MOF glass coating, except that the surface became much smoother compared with pristine graphite particles (Fig. 2h and j, Figs S11–S18). Notably, the ultra-thin MOF glass coating layer (short-range ordered) of the 2 wt% glass@graphite can be clearly observed, with a thickness of approximately 5–8 nm (Fig. 2i and k). The uniform distribution of Zn, P, N and O elements across glass@graphite materials signified the homogeneous coating of the MOF glass coating (Fig. 2l, Figs S14, S16 and S18). These results together verified that the graphite particles were completely coated by a crack-free and ultra-thin MOF glass layer.

### Fast Li-ion desolvation and diffusion of the glass@graphite anode

**Figure 3.**
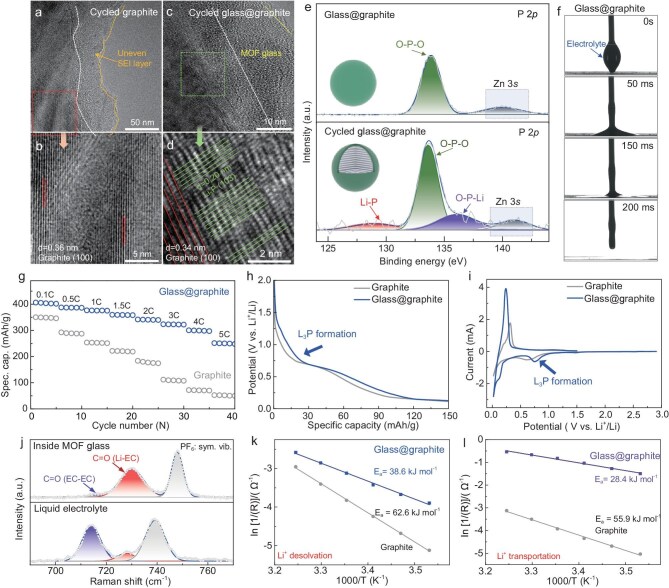
Excellent fast-charging performance of glass@graphite electrode. Scanning electron microscopy (SEM) and high-resolution TEM (HRTEM) images of (a and b) cycled graphite and (c and d) cycled glass@graphite. (e) High-resolution XPS spectra of P 2p for glass@graphite before and after cycling, respectively. (f) Contact angles of the electrolyte (LiPF_6_ in EC:DMC:EMC at 1 : 1 : 1 vol%) on glass@graphite electrode. (g) Rate performance of graphite//Li half-cell and glass@graphite//Li half-cell (with 2 wt% MOF glass coating). (h) Initial discharge curves of graphite//Li half-cell and glass@graphite//Li half-cell and (i) the corresponding CV curves. (j) Raman spectrum of liquid electrolyte and electrolyte formed inside MOF glass. Comparison of (k) desolvation and (l) transportation activation energies for lithium ions.

To further evaluate the advantage of the crack-free, ultra-thin glass coating on graphite, glass@graphite//Li half-cells were under electrochemical measurement alongside pristine graphite//Li half-cells for comparison. Firstly, both the cycled graphite and glass@graphite were under investigation. Clearly, for the cycled graphite, a thick and uneven SEI layer (30–50 nm) was formed on its surface, and the interlayer spacing expanded from an original 0.34 nm to 0.36 nm (Fig. 3a and b). The decomposition of electrolyte led to the thick SEI layer, while the co-intercalation of solvated lithium ions led to the expanded graphite interlayer spacing. In sharp contrast, the cycled glass@graphite electrode exhibited no detectable byproducts and the graphite interlayer spacing was preserved at 0.34 nm (Fig. 3c and d), which was attributable to its electrically insulating, uniform MOF glass coating that prevents parasitic reactions. Interestingly, after cycling, a new phase emerged between the MOF glass layer and bulk graphite (highlighted by green lines), exhibiting a significantly narrower interlayer spacing of 0.2 nm, attributed to the (103) crystal plane of Li_3_P [40–42]. The formation of Li_3_P composite was further verified by the corresponding X-ray photoelectron spectroscopy (XPS) results shown in Fig. 3e. Specifically, compared with that of the newly prepared uncycled glass@graphite particles, which only demonstrated one O–P–O peak (located at 133.7 eV, ascribed to MOF glass), the high-resolution P 2p XPS spectrum of the cycled glass@graphite showed two newly prominent peaks (Fig. 3e). The two peaks, situated about 129.0 and 136.1 eV, can be ascribed to Li–P and O–P–Li bonds, respectively, both of which were related to the formation of Li_3_P. The etching XPS analysis effectively verified the dynamic changes in this double-layer structure (Fig. S19). The newly formed Li_3_P exhibited a strong affinity for lithium ions, simultaneously facilitating their desolvation and transport, making it an exemplary lithium-ion superconductor and beneficial for fast charging [40]. Compared with that of the graphite electrode (Fig. S20), the newly prepared glass@graphite electrode was quickly wetted with the liquid electrolyte [1 M LiPF_6_ in ethylene carbonate/dimethyl carbonate/ethyl methyl carbonate (EC/DMC/EMC)], which was also one of the important factors in accelerating fast charging (Fig. 3f). Cycling performance under 0.5 and 1 C (Fig. S21) revealed 2 wt% glass@graphite as the optimal formulation. This selection aligns with prior conclusions derived from coating thickness analysis. To validate the hypothesis of fast charging, rate performance was evaluated in half-cell configurations (glass@graphite//Li vs graphite//Li). As shown in Fig. 3g, the graphite//Li half-cell displayed poor rate performance during charging/discharging from 0.1 to 5 C, particularly under high current densities exceeding 2 C. When cycled at 4 and 5 C, it delivered an exceptionally low capacity of less than 100 mAh/g. In stark contrast, the glass@graphite//Li half-cell demonstrated outstanding rate performance, delivering ultra-high capacities exceeding 300 and 250 mAh/g under 4 and 5 C—over five times higher than those of the graphite//Li half-cell, respectively (Fig. 3g). It is worth noting that these obtained electrochemical performances demonstrate significant advancements compared to previously reported results in this field (Table S2). The platform region near about 0.75 V (vs Li^+^/Li) in the charge–discharge curve (Fig. 3h and Fig. S22) of the glass@graphite//Li half-cell and the reduction peak at the corresponding potential in the cyclic voltammetry (CV) curve (Fig. 3i and Fig. S23) were both attributed to the formation of the lithium ion-accelerating Li_3_P phase during the first discharge process of batteries. We also found that electrolyte inside the MOF glass layer of the cycled glass@graphite was more aggregative than that of the typical liquid electrolyte added during battery assembling, as the Li–EC-related peak (red curve) within the MOF glass layer was much stronger than the EC–EC-related peak (purple curve) (Fig. 3j). The highly aggregated electrolyte was attributed to the narrow 2.93 Å pore windows of the MOF glass, which effectively facilitated lithium-ion pre-desolvation, as illustrated in Fig. 1b. Given that the solvated lithium ions in commercial electrolytes were significantly larger than the subnanopores of the MOF glass, they must shed most of their solvent molecules to enter the glass channels. This process resulted in a highly aggregated electrolyte composed of partially desolvated lithium ions with significantly smaller sizes, which was beneficial for fast lithium-ion diffusion. To further verify our conjecture, both the activation energies of lithium-ion desolvation and transportation were studied. Clearly, as shown in Fig. 3k and l, the glass@graphite//Li half-cell demonstrated significantly lower lithium-ion desolvation energy (38.6 vs 62.6 kJ/mol) and transport activation energy (28.4 vs 55.9 kJ/mol) compared to the graphite//Li half-cell. The 2.93 Å MOF glass enabled easy lithium-ion desolvation, generating smaller, partially desolvated lithium ions, while the Li_3_P component, with its strong affinity for these ions, further accelerated their rapid diffusion. These combined factors significantly enhanced the fast-charging performance of the glass@graphite anode. Our MOF glass coating strategy achieved dual functionality by simultaneously promoting Li⁺ pre-desolvation and *in situ* Li₃P generation to enhance ion conduction—a capability unattainable with conventional MOF or Li₃P coatings, which only address either pre-desolvation or transport of Li^+^ [38,40].

### Glass@graphite anode promoting stable LiF-rich SEI and inhibiting Li dendrites

**Figure 4.**
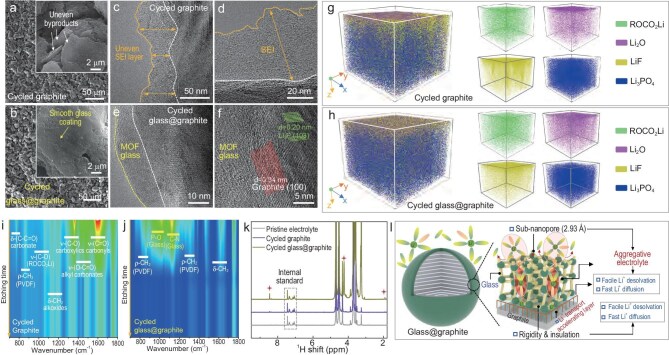
Characterizations of the cycled graphite and cycled glass@graphite. Scanning electron microscopy (SEM) and TEM images of (a, c and d) cycled graphite and (b, e and f) cycled glass@graphite. 3D views of elements distribution in the TOF-SIMS sputtered volumes of (g) cycled graphite and (h) cycled glass@graphite, and the corresponding color mappings of (i) cycled graphite and (j) cycled glass@graphite based on etching FTIR spectra. (k) NMR results of cycled graphite//Li half-cell and glass@graphite//Li half-cell. (l) Schematic representation of the MOF glass coating facilitating easy lithium-ion pre-desolvation and accelerating fast lithium-ion transport of the glass@graphite electrode.

The beneficial effects of the MOF glass coating were further confirmed through analysis of the cycled glass@graphite after extended cycling for 200 cycles. Comparison of the morphologies revealed a stark contrast; the cycled graphite electrodes were covered with unevenly distributed byproducts (Fig. 4a and Fig. S24), whereas the cycled glass@graphite electrodes showed a clean surface, free from noticeable byproducts (Fig. 4b and Fig. S25). Furthermore, high-resolution transmission electron microscopy (TEM) images revealed the formation of a thick and uneven SEI layer on the cycled graphite particles (Fig. 4c and d, Fig. S26). In stark contrast, the cycled glass@graphite particles remained a uniform MOF glass on the graphite surface, identical to their initial cycled state, with no byproducts observed (Fig. 4e and Fig. S27). More importantly, the graphite in cycled glass@graphite maintained its original 0.34 nm interlayer spacing throughout extended cycling, without detectable byproduct formation. Significantly, the electrochemically formed Li₃P component persisted through multiple lithium-ion insertion/extraction cycles (Fig. 4f and Fig. S27), highlighting exceptional electrochemical stability of this critical layer. Time-of-flight secondary ion mass spectrometry (TOF-SIMS) was also employed to further study the surface component/structural information of two cycled electrodes. For the cycled graphite electrode, depth-profiled TOF-SIMS data revealed a thick and uneven distribution of organic species and inorganic moieties on the surface (Fig. 4g and Fig. S28a), indicating extensive byproduct formation caused by electrolyte decomposition (EC/DMC/EMC). In contrast, the cycled glass@graphite electrode exhibited no obvious electrolyte decomposition-related byproducts, which benefited from the electrically insulating MOF glass layer (Fig. 4h and Fig. S28b). The depth-resolution etching FTIR technique was then employed to obtain more detailed information about the surface information of the two cycled samples. For the cycled graphite, multiple strong peaks associated with byproducts were clearly observed (Fig. 4i and Fig. S29a). Specifically, the sharp peaks, corresponding to carboxyl (C–O), carbonyl (C=O) and alkyl carbonate (ROCO_2_Li), were primarily derived from the decomposition of electrolyte. These peaks persisted throughout the entire etching process, further confirming the presence of a thick byproduct layer coating on the surface of the cycled graphite electrode. A different result was obtained for the cycled glass@graphite electrode. For the cycled glass@graphite, only peaks associated with the MOF glass and weak signals from the polyvinylidene fluoride (PVDF) binder can be detected during the initial etching process, while no peaks related to decomposition products of electrolyte can be observed (Fig. 4j and Fig. S29b), which was consistent with the TOF-SIMS results. The corresponding nuclear magnetic resonance (NMR) results of the two electrodes were also recorded. Compared with the cycled graphite electrode, which demonstrated serious electrolyte decomposition, the cycled glass@graphite electrode did not exhibit any electrolyte decomposition-related peaks (Fig. 4k, red stars). In addition, the glass@graphite//Li half-cell also demonstrated much lower resistance before and after electrochemical cycling processes (Fig. S30). Specifically, prior to electrochemical cycling, the MOF glass coating on the glass@graphite composite significantly enhanced electrolyte wettability (Fig. 3f and Fig. S20), resulting in measurably lower interfacial resistance in glass@graphite//Li half-cells versus unmodified graphite//Li counterparts (Fig. S30a). During cycling, the MOF glass facilitated lithium-ion pre-desolvation at the graphite interface and promoted highly concentrated electrolyte within its nanochannels. These effects—combined with the formation of Li₃P species—synergistically enhanced lithium-ion transport kinetics and fostered an anion-derived SEI layer. This led to the much lower resistance of the glass@graphite//Li half-cells. This synergistic effect resulted in reduction in total cell resistance for glass@graphite//Li half-cells compared to unmodified graphite cells, as quantified by electrochemical impedance spectroscopy (Fig. S30b and c). Furthermore, the EIS result also confirmed the effectiveness of the MOF glass in protecting the graphite anode by preventing structural degradation and suppressing side reactions. Based on results obtained from Figs 2–4, we summarized the specific positive role of the MOF glass coating in protecting the graphite electrode, as schematically illustrated in Fig. 4l. Specifically, the subnanopore structure of the outer layer of the MOF glass promoted easy pre-desolvation of solvated lithium ions (dropped most of the electrolyte solvents) before they reached the surface of the graphite particles, and then generated highly aggregative electrolyte inside the narrow glass channel of the MOF glass. Consequently, the inner layer, composed of Li_3_P, a super-Li^+^ conductor, promoted the fast transportation of partially desolvated lithium ions with much smaller sizes. Furthermore, the rigid and electrically insulating MOF glass prevented the decomposition of electrolyte, thus contributing to the formation of a stable LiF-rich SEI and inhibiting Li dendrites. By implementing this perfect particle-level pre-desolvation method, the fast-charging performance and long-term cycle stability of graphite can be significantly improved.

### Enhanced cycling stability and energy density

**Figure 5.**
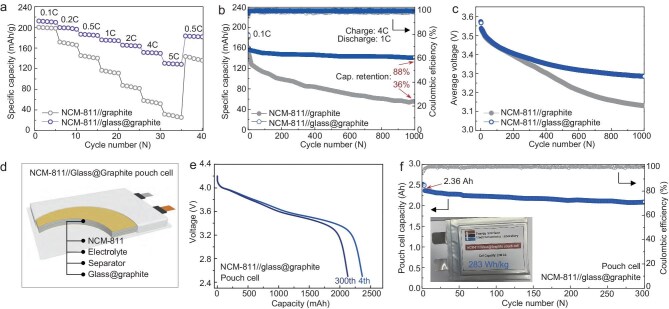
Electrochemical performances of full cells and pouch-cell performance based on NCM-811//glass@graphite. (a) Rate performance, (b) cycling performance and (c) average voltage comparison of assembled NCM-811//graphite and NCM-811//glass@graphite full cells. (d) Schematic illustration of the NCM-811//glass@graphite pouch-cell structure. (e) The comparative capacity–voltage curves of the 4th and 300th cycles for the NCM-811//glass@graphite pouch cell, and the corresponding (f) cycling performance of NCM-811//glass@graphite pouch cell (inset, digital photo of the pouch cell).

To further verify the structural advantages and utilization prospect of glass@graphite, NCM-811//glass@graphite full cells and a pouch cell were assembled by using NCM-811 as the cathode and glass@graphite as the anode. Firstly, the rate performances (from 0.1 to 5 C) of both NCM-811//glass@graphite and NCM-811//graphite were evaluated. The NCM-811//graphite half-cell experienced very fast capacity decay after 0.2 C current rate, and delivered only 35 mAh/g capacity at 5 C (Fig. 5a, grey circles). For sharp contrast, the NCM-811//glass@graphite full cell demonstrated a remarkably enhanced rate performance, delivered a high capacity of 165 mAh/g at 2 C, and preserved 155 mAh/g at 4 C. Even cycled under a high current rate of 5 C, it still exhibited a high capacity of nearly 135 mAh/g (Fig. 5a, purple circles), four times higher than that of the NCM-811//graphite full cell under the same conditions. The NCM-811//glass@graphite full cell exhibited outstanding fast-charging performance even under the demanding condition of charging at 4 C and discharging at 1 C (Fig. 5b). Specifically, after being cycled for 1000 cycles, the NCM-811//glass@graphite full cell still retained above 140 mAh/g specific capacity, corresponding to 88% capacity retention (calculated from the fourth circle, Fig. 5b, blue circles). In contrast, the NCM-811//graphite full cell sustained merely 60 mAh/g, with a very low 36% capacity retention (Fig. 5b, grey circles). In addition to the significantly improved fast-charging performance, the NCM-811//glass@graphite full cell also demonstrated much smaller average voltage fluctuations during cycling compared with the NCM-811//graphite full cell (Fig. 5c), indicating superior capacity reversibility and enhanced cycle stability of glass@graphite. All of these inspiring results were attributed to the advantages from easy pre-desolvation of lithium ions and accelerated lithium-ion transport induced by the MOF glass. Smaller average voltage changes during cycles also supported that conclusion. It is also worth noting that the strategy reported in this work is exceptionally favorable for large-scale manufacturing. Unlike conventional coating methods, which often demand high temperatures or complex conditions (Table S3), our approach offers several key advantages, including low-temperature processing (175°C), rapid coating time (30 min), non-toxic and environmentally friendly chemistry, and cost-effectiveness. These benefits collectively position our strategy as a highly scalable and industrially viable solution. Inspired by these afore-obtained results, an NCM-811//glass@graphite pouch cell was fabricated and measured (Fig. 5d). The prepared NCM-811//glass@graphite pouch cell delivered a high initial capacity of 2.36 Ah and ultra-high energy density of 283 Wh/kg (calculated from the fourth cycle), maintaining over 80% of its capacity even after a long cycling life of 300 cycles (Fig. 5e and f). Together, these inspiring electrochemical results highlighted the strong potential of MOF glass in enhancing both the fast-charging performance and long-term cycle stability of graphite anodes.

## CONCLUSION

In summary, by uniformly coating the graphite with glassy MOF with 2.93 Å pore windows under a low temperature of 175°C for 30 min, we simultaneously facilitated easy lithium-ion pre-desolvation and accelerated lithium-ion transport during electrochemical cycling process. After the initial discharge, the MOF glass-coated graphite (glass@graphite) developed a distinct double-layer structure. The outer layer, made of electron-insulating MOF glass with rigid 2.93 Å pores, promoted efficient lithium-ion pre-desolvation and facilitated the formation of a highly concentrated electrolyte with compact partially desolvated lithium ions within the MOF glass channels. The inner section, in direct contact with graphite, formed an Li_3_P component-containing layer that enhanced rapid lithium-ion transport. The smaller, partially desolvated lithium ions formed inside the MOF glass channels easily passed through this Li_3_P-containing layer, further accelerating lithium-ion diffusion. This unique double-layered structure effectively contributed to the formation of a stable LiF-rich SEI, prevented electrolyte decomposition-related side reactions and the formation of Li dendrites, and enabled exceptional fast-charging performance of glass@graphite (delivering ultra-high capacities exceeding 300 mAh/g and 250 mAh/g under 4 and 5 C, respectively.). As a result, the NCM-811//glass@graphite full cell exhibited exceptional fast-charging performance with capacity retention at 88% after 1000 cycles at a high rate of 4 C. More realistically, the NCM-811//glass@graphite pouch cell achieved a high energy density of 283 Wh/kg at 2.36 Ah, retaining 80% of its capacity after 300 cycles. This MOF glass coating strategy opens new avenues for developing fast-charging anode materials and offers valuable insights for advancing next-generation high-energy-density LIB technologies.

## MATERIALS AND METHODS

### Synthesis of Zn–P–dmbIm MOF powder

Zinc acetate dihydrate (439 mg, 2 mmol), 5,6-dimethylbenzimidazole (584.8 mg, 4 mmol) and phosphoric acid (420 μL, 6 mmol) were placed in a mortar and manually ground for 15 min. Finally, the powder was washed with dichloromethane three times and dried at 70°C for 10 h, and then Zn–P–dmbIm MOF powder was successfully prepared.

### Synthesis of MOF glass and MOF glass film

The prepared Zn–P–dmbIm MOF powder was heated to 175°C and held for 30 min under an inert atmosphere, and finally cooled to room temperature. After that, the MOF glass was obtained. As for the MOF glass film, mechanical pressure was exerted to urge Zn–P–dmbIm MOF powder to turn into sheets (diameter was 19 mm) under 2 MPa, and other operations remained the same.

### Synthesis of glass@graphite anode

The pristine graphite anode was purchased from Dongguan Kelude Innovation Technology Co., Ltd. First, the pristine graphite was mixed with Zn–P–dmbIm MOF powder in a mortar and ground for 10 min. Second, the mixture was heated to 175°C in an inert atmosphere for 30 min before sharp cooling to room temperature (25°C). Finally, the anode coated by MOF glass was obtained successfully, all of which was denoted as glass@graphite. The weight ratio of the Zn–P–dmbIm MOF powder and pristine graphite was different, such as 2 wt%, 5 wt% and 10 wt%, correspondingly denoted as 2 wt% glass@graphite, 5 wt% glass@graphite and 10 wt% glass@graphite.

### Electrode preparation, cell assembly and electrochemical measurements

The weight ratio was 8 : 1 : 1 of active materials (pristine graphite or as-prepared glass@graphite), carbon black and PVDF powder, uniformly mixed in *N*-methyl-2-pyrrolidone (NMP). The uniform slurry was then pasted onto Cu foil and dried in a vacuum oven at 80°C for 12 h. The mass loading of active materials for working electrodes was about 1.2–1.5 mg (diameter of 12 mm). CR2032 coin cells, according to the order of negative shell, shrapnel, spacer, anode, separator, cathode and positive shell, were assembled in an argon-filled glovebox with both the moisture and oxygen content lower than 0.01 ppm. 1 M LiPF_6_ in EC/DMC/EMC (1 : 1 : 1 vol%) was used for all those coin cells. For the preparation of the LiNi_0.8_Co_0.1_Mn_0.1_O_2_ (NCM-811) electrode, the weight ratio was 9 : 0.5 : 0.5 of NCM-811, carbon black and PVDF powder, uniformly mixed in NMP. The uniform slurry was then pasted onto Al foil and dried in a vacuum oven at 80°C for 12 h. The mass loading of the NCM-811 cathode for coin cells was about 5.5 mg (diameter of 12 mm). The mass loading of the NCM-811 cathode for the pouch cell was about 32.0 mg/cm^2^. The pouch cell was assembled by using NCM-811 as the cathode (5 × 10 cm^2^) and glass@graphite as the anode (5 × 10 cm^2^), and the negative capacity/positive capacity ratio (N/P ratio) was 1.10. Both the prepared NCM-11//graphite and NCM-11//glass@graphite coin cells, and the NCM-11//glass@graphite pouch cell were operated with a potential limit between 2.5 and 4.2 V. It is worth noting that the prepared NCM-11//glass@graphite pouch cell was measured under external pressure. An enclosure for applying stack pressure was used, where pouch cells are uniaxially constrained in a steel enclosure with an adjustable wall, which can be tightened to apply varying uniaxial pressures (the stack pressure was about 10 MPa). The galvanostatic electrochemical measurements were carried out under potential control using the LAND battery test system at room temperature (25°C in a climatic chamber).

### Characterizations

See Supplementary Data for details.

## Supplementary Material

nwaf349_Supplemental_File
