# Supplementary material for: Accelerating lithium-ion pre-desolvation and transport via glassy MOF for fast-charging and high-energy-density lithium-ion batteries
Source: Natl Sci Rev. 2025 Aug 22;12(11):nwaf349. doi: 10.1093/nsr/nwaf349 (PMC12547410; doi:10.1093/nsr/nwaf349)
Supplement: nwaf349_Supplemental_File [file nwaf349_supplemental_file.pdf]

Supplementary Data

Accelerating lithium-ion pre-desolvation and transport via glassy MOF for fast-charging and high-energy-density lithium-ion batteries

Yan Xu[1], Danni Zhang[1], Shibin Zhang[1], Lishun Bai[1], Yue Liu[1], Jingwen Zhao[2], Zhi Chang[1,]* and Haoshen Zhou[2,]*

[1]School of Materials Science and Engineering, Key Laboratory of Electronic Packaging and Advanced Functional Materials of Hunan Province, Central South University, Changsha 410083, China.

[2]Center of Energy Storage Materials & Technology, College of Engineering and Applied Sciences, Jiangsu Key Laboratory of Artificial Functional Materials, National Laboratory of Solid State Micro-structures, and Collaborative Innovation Center of Advanced Micro-structures, Nanjing University, Nanjing 210093, China.

*Corresponding authors. E-mails: zhichang@csu.edu.cn; hszhou@nju.edu.cn.

Experimental Procedures

Synthesis of Zn-P-dmbIm MOF powder

Zinc acetate dihydrate (439 mg, 2 mmol), 5,6-dimethylbenzimidazole (584.8 mg, 4 mmol), and phosphoric acid (420 μL, 6 mmol) were placed in a mortar and manually ground for 15 min. Finally, washing the powder with dichloromethane three times and dried at 70 ℃ for 10 h, and then Zn-P-dmbIm MOF powder was successfully prepared[1].

Synthesis of MOF Glass and MOF Glass Film

Heating the prepared Zn-P-dmbIm MOF powder until 175 ℃ and holding 30 min under inert atmosphere, and finally cooling to room temperature. After that, the MOF Glass was obtained. As for MOF Glass Film, mechanically pressure was exerted to urge Zn-P-dmbIm MOF powder turn into sheets (diameter was 19 mm) under 2 MPa, and other operations remained the same.

Synthesis of Glass@Graphite anode

The Pristine Graphite anode was purchased from Dongguan Kelude Innovation Technology Co., Ltd. First, the Pristine Graphite was mixed with Zn-P-dmbIm MOF powder in a mortal and ground for 10 min. Second, the mixture was heated under 175 ℃ in inert atmosphere for 30 min before sharp cooling to room temperature (25 ℃). Finally, the anode coated by MOF Glass was obtained successfully, all of which denoted as Glass@Graphite. Weight ratio of Zn-P-dmbIm MOF powder and Pristine Graphite was different, such as 2 wt%, 5 wt% and 10 wt%, correspondingly denoted as 2 wt% Glass@Graphite, 5 wt% Glass@Graphite and 10 wt% Glass@Graphite.

Electrodes Preparation and batteries assembly

The weight ratio was 8:1:1 of active materials (Pristine Graphite or as-prepared Glass@Graphite), carbon black and polyvinylidene fluoride (PVDF) powder, uniformity mixing in N-methyl-2-pyrrolidone (NMP). Then the uniform slurry was pasted onto Cu foil and dried in vacuum oven at 80 ℃ for 12 h. The mass loading of active materials for

working electrodes was about 1.2-1.5 mg (diameter of 12 mm). CR2032 coin-cells, according to the order of negative shell, shrapnel, spacer, anode, separator, cathode and positive shell, were assembled in an argon-filled glovebox with both the moisture and oxygen content lower than 0.01 ppm. 1 M $LiPF_6$ in ethylene carbonate (EC)/dimethyl carbonate (DMC)/ethyl methyl carbonate (EMC) (1:1:1 vol%) was used for all those coin-cells. The pouch-cell was assembled by NCM-811 cathode (5 × 10 cm^2) and Glass@Graphite anode (5 × 10 cm^2).

Characterizations

Morphology and structure characterization

A homemade H-type cell with transparent dimethoxyethane (DME, right), a DME solution containing dissolved polysulfides (left, dark red), and the prepared MOF Glass (middle, as separator) was fabricated to investigate the polysulfide permeation test of the MOF Glass. X-ray diffraction (XRD) was investigated by a Rigaku Mini Flex 600 diffractometer fitted with Cu K_α-radiation (λ=1.5418 Å) to identify the structure information. Morphology images of every experimental sample were characterized by scanning electron microscopy (SEM, MIRA4 LMH) equipped with X-ray energy dispersion spectroscopy (EDS, Ultim Max 40), and high-resolution transmission electron microscopy (HR-TEM, JEM-F200). Thermogravimetric analysis (TGA) and Differential scanning calorimetry (DSC) measurements DSC was carried out on thermal analyzer (NETZSCH, STA 409) from 30 to 300 ℃ at the rate of 5 ℃ min^{-1} under dry flow of Ar. The Zn-P-dmbIm MOF powder was pelleted into a cylinder (radius of 1.5 cm, thickness of ~1 mm), and the MOF Glass was prepared into a film (larger than 1 × 1 cm^2 square, thickness of ~1 mm), to execute Positron annihilation lifetime spectroscopy (PALS) test. The PALS measurements, using ^{22}Na to be positrons source, were carried out at 25 ℃ and the experimental data were analyzed by a four-finite lifetime component using LT 9.0. The molecule structure was obtained from nuclear magnetic resonance (NMR) spectroscopy characterizations (500 MHz Ultra-ShieldTM, Bruker), and attenuated total reflection Fourier-transform infrared (ATR-FTIR) measurements using FT/IR-6200 spectrometer (JASCO Corp.). X-ray photoelectron spectroscopy (XPS) characterization was carried out using VG scientific ESCALAB 250 spectrometers with monochromic Al Kα source (1486.6 eV) under ultra-high vacuum. Raman spectra were performed using JASCO microscope spectrometer (NRS-1000DT). TOF-SIMS analysis was completed

by a TESCAN LYRA3 GM scanning electron microscope equipped with TOF-SIMS accessories, and the measuring beam was 1 nA.

Electrochemical measurements

The galvanostatic charging/discharging test were conducted on LAND battery test system CT3002A (5V1mA&10mA8C1U), operating at a potential interval between 0.02-1.5 V. Cyclic voltammetry (CV) curves and electrochemical impedance spectroscopy (EIS) results were obtained from an CORRTEST electrochemical workstation (CS Studio6, CS350M in COM3).

The calculation formula of the activation energies

The activation energies of lithium-ion desolvation and transportation were studied from electrochemical impedance spectroscopy (EIS) under different temperature of 10 ^oC, 15 ^oC, 20 ^oC, 25 ^oC, 30 ^oC, 35 ^oC, respectively. The activation energies were calculated by fitting the EIS curves at different temperatures.

$$-lnR_{sei} = -\frac{E_a}{RT} + lnA$$

$$-lnR_{ct} = -\frac{E_a}{RT} + lnA$$

Where R_{sei} is the resistance of the Li^+ transportation, R_{ct} is the resistance of the Li^+ desolvation process, R is the universal gas constant (8.314 J mol^{-1} K^{-1}), T is the absolute temperature (K), and A is the pre-exponential factor. Taking 1000/T as the horizontal coordinate and -ln (R_{ct}) as the vertical coordinate, the opposite of the slope of the straight line obtained by plotting the fit is the value of E_a.

Supplementary Figures

Table S1. PALS results for Zn-P-dmbIm MOF powder and the MOF glass.

Sample	t_3 (ns)	I_3 (%)	Diameter (nm)	t_3 (ns)	I_3 (%)	Diameter (nm)
Zn-P-dmbIm powder	1.021	21.7	0.340	1.917	5.05	0.552
Glass	0.895	12.3	0.293	1.582	14.6	0.482

Table S2. Comparison in electrochemical performance of this work and others

**Sample**	**Rate, Cycle number, Capacity retention, Initial CE**	**Rate, Maximum rate capacity**	**Ref.**
Glass@Graphite	0.5 C, 200, 75%, 99%	5 C, 275 mAh/g	This work
EGC	0.2 C, 200, 86.64%, 99%	5 C, 200 mAh/g	2
TiO_{2-x}-decorated graphite	0.1 C, 100, 98.5%, 76.9%	5 C, ~270 mAh/g	3
Turbostratic carbon-coated graphite	1 C, 300, 89.66%, 87%	3 C, 150 mAh/g	4
VGSs/Graphite	0.2 C, 100,85.3%, 107.8%	5 C, 170 mAh/g	5
CTP76-coated graphite	1 C, 50, 89.7%, 90.0%, ≈96%	5 C, 235 mAh/g	6
Graphite etching	2.5 C, 100, /, 96.7%	6 C, 140 mAh/g	7
G@C	1 C, 400, 87.5%, 97.5%	3 C, ≈75 mAh/g	8
GDY@graphite	1 C, 250, 82%, 93.5%	5 C, ≈65 mAh/g	9

Table S3. Comparative statistical of MOF Glass and other coating materials

Sample	Temperature/^{o}C	Synthesis time/(h)	Toxicity	Ref.
Glass@Graphite	175	0.5	Low	This work
PLA@PDA-ZIF	/	2	Medium	10
Si@C@ZIF-67-800N	800	3	High	11
pSiMS@C	280	72	Medium	12
u-NCM62	600	6	Medium	13
Cobalt ZIF-62 glass	460	0.8	Low	14

Figure S1. Structural schematic of Zn-P-dmbIm MOF powder.

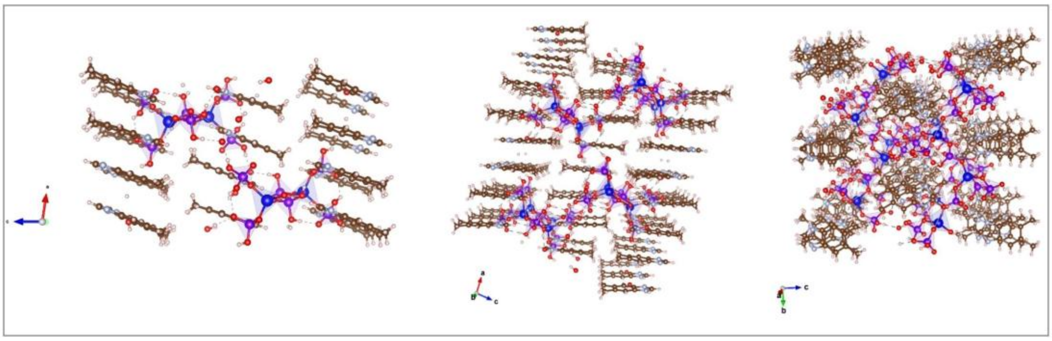

Figure S2. Schematic illustration of preparation process of (a) the Zn-P-dmbIm MOF powder[1] and (b) the Glass@Graphite anode.

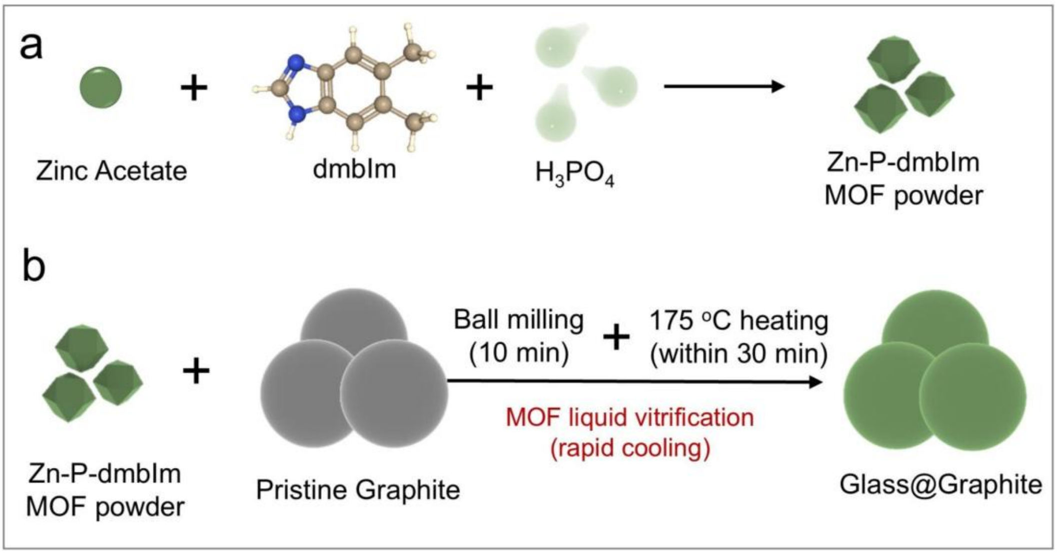

Figure S3. (a) TGA and (b) DSC curves of Zn-P-dmbIm MOF powder.

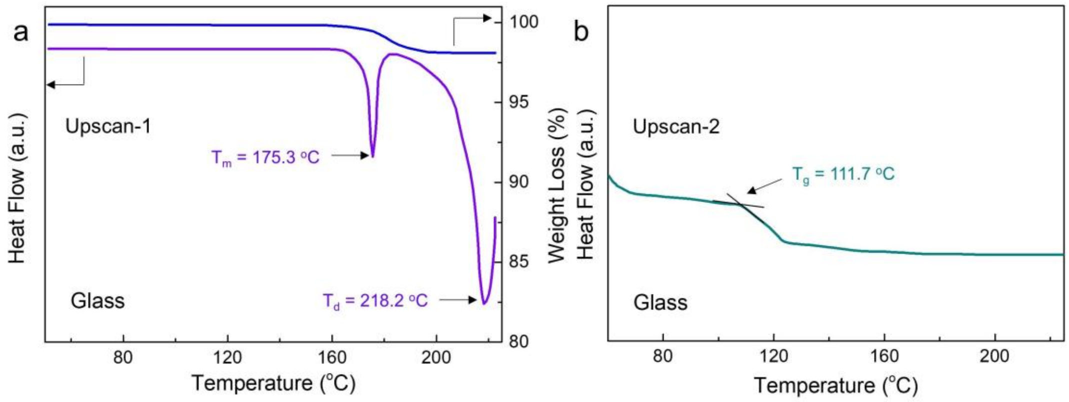

Figure S4. SEM images of Zn-P-dmbIm MOF powder.

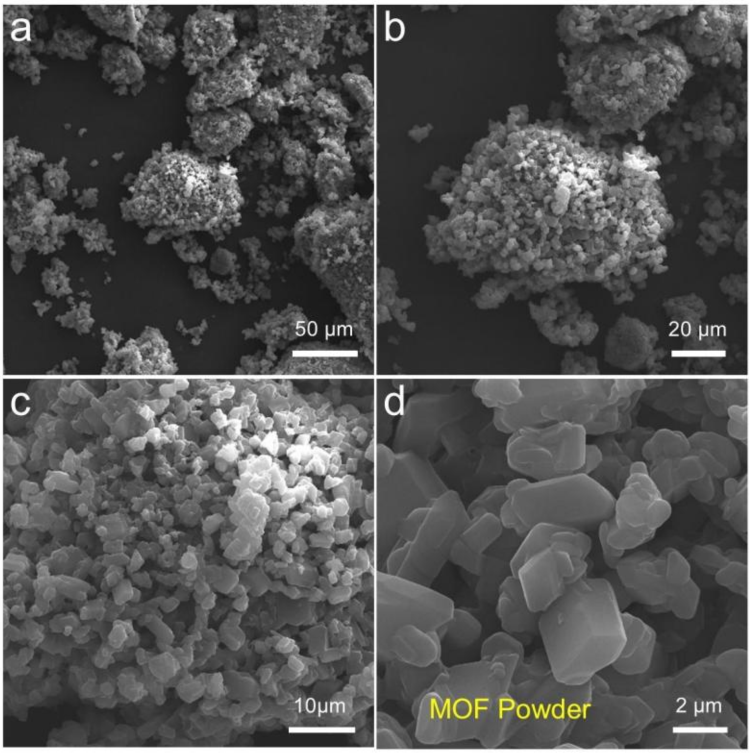

Figure S5. SEM images of MOF glass film.

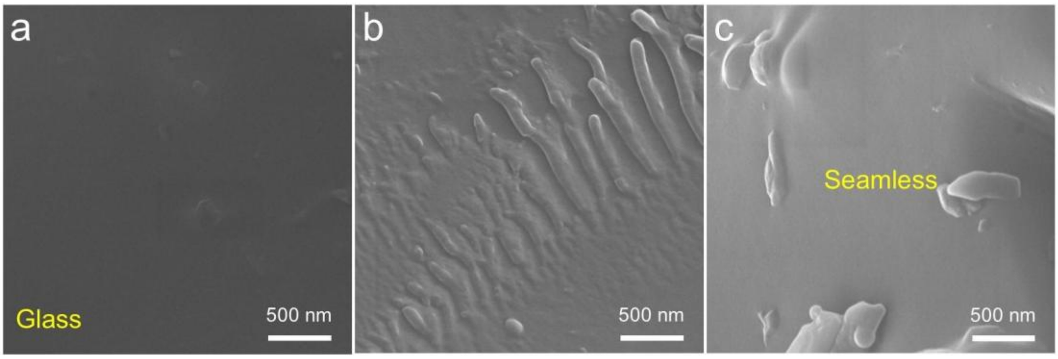

Figure S6. TEM images of Zn-P-dmbIm MOF powder.

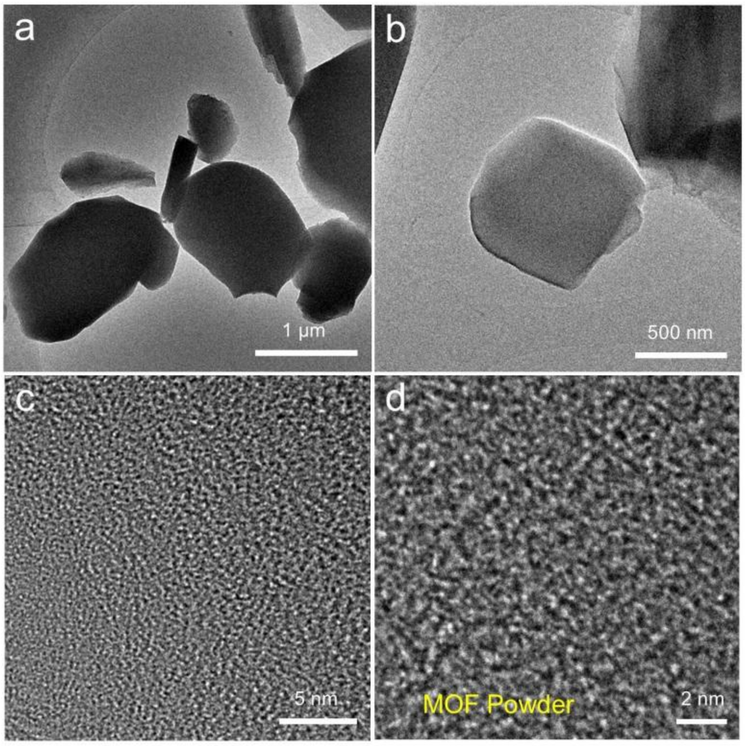

Figure S7. EDS elemental mapping images of Zn-P-dmbIm MOF powder.

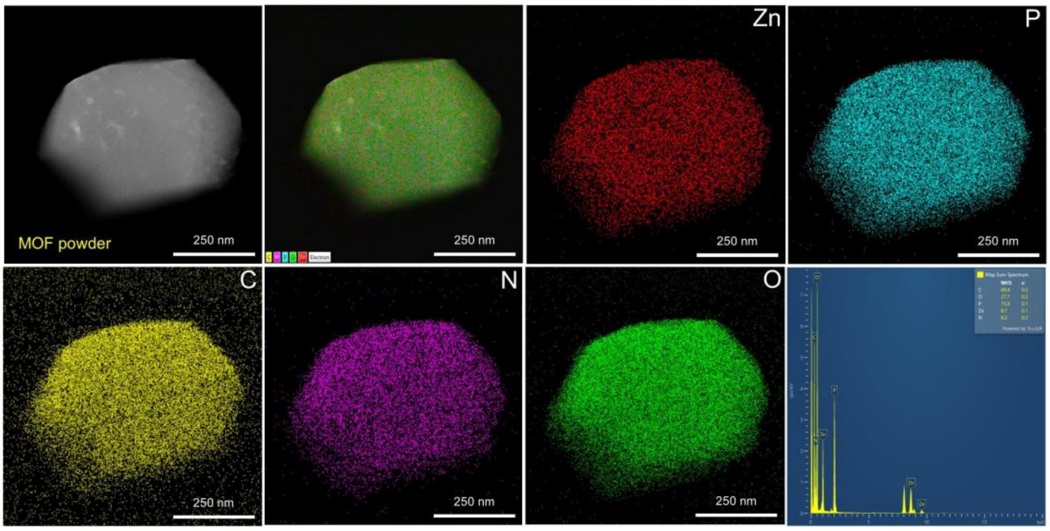

Figure S8. TEM images of MOF glass.

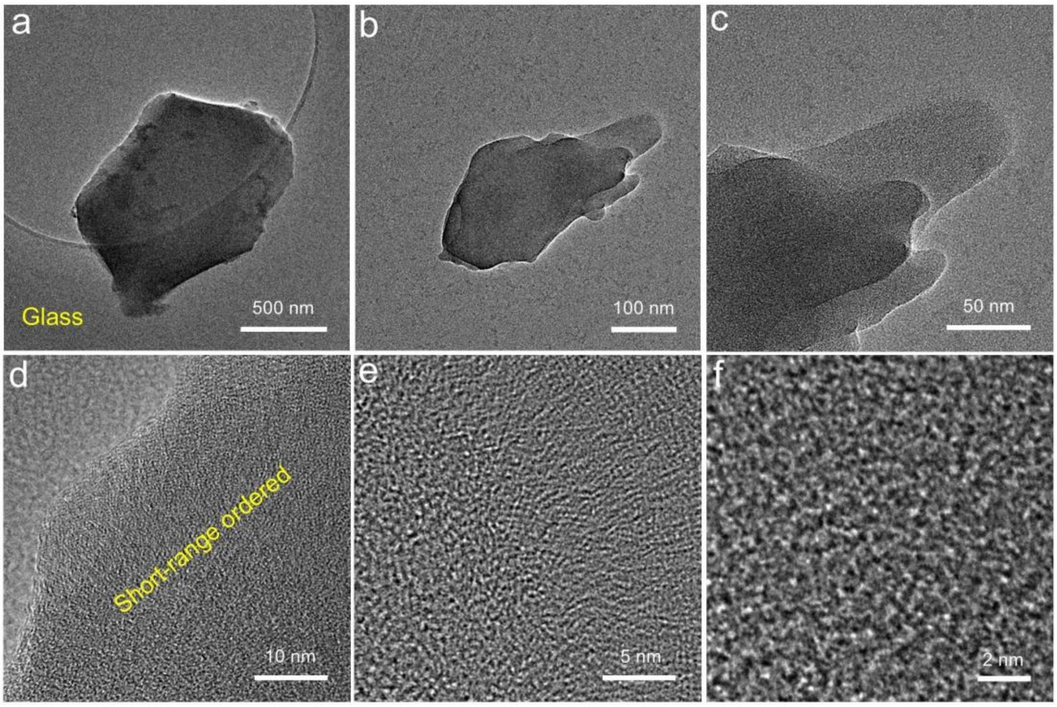

Figure S9. EDS elemental mapping images of MOF Glass.

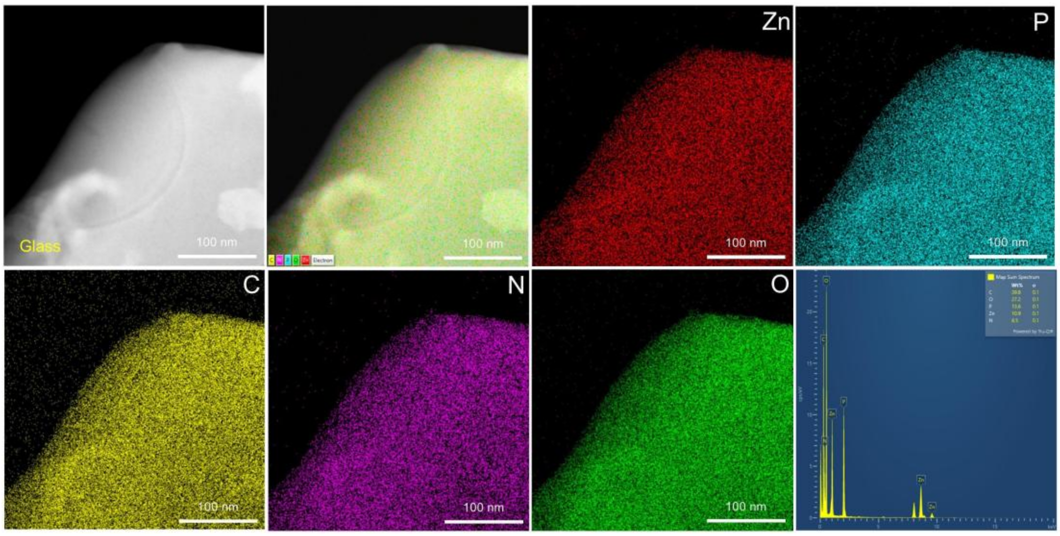

Figure S10. ATR-FTIR spectra of Zn-P-dmbIm MOF powder and MOF glass.

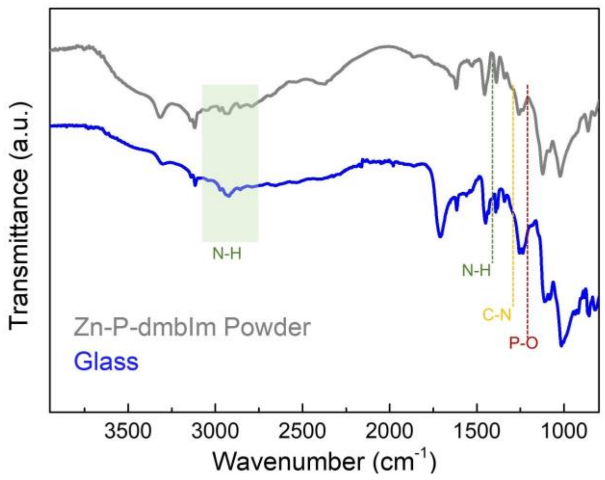

Figure S11. XRD of Pristine Graphite, and Glass@Graphite with different Glass coating proportions, including 2 wt% Glass@Graphite, 5 wt% Glass@Graphite, 10 wt% Glass@Graphite.

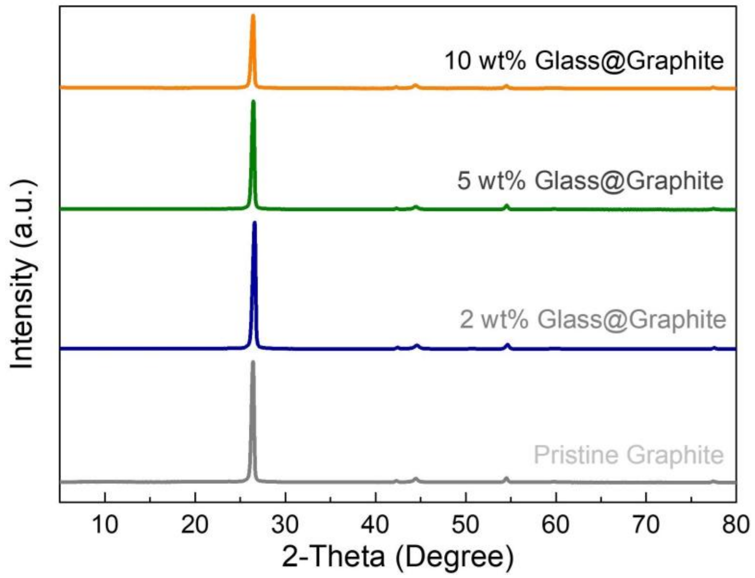

Figure S12. (a, b) SEM and (c) TEM images of Pristine Graphite.

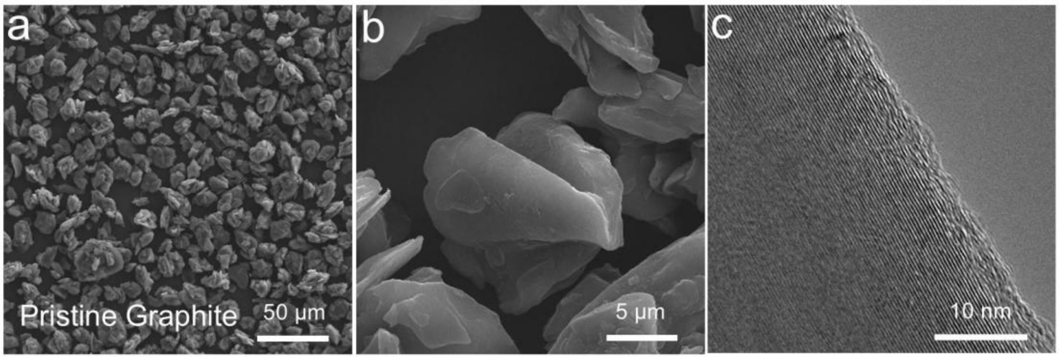

Figure S13. (a-c) SEM and (d-f) TEM images of 2 wt% Glass@Graphite.

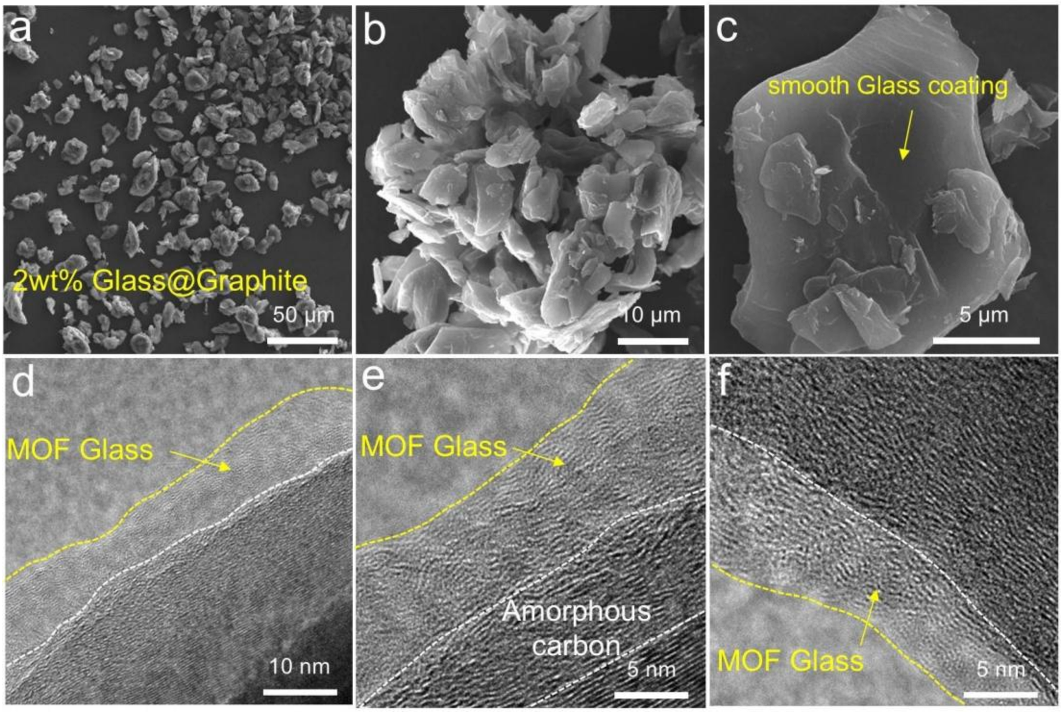

Figure S14. EDS elemental mapping images of 2 wt% Glass@Graphite.

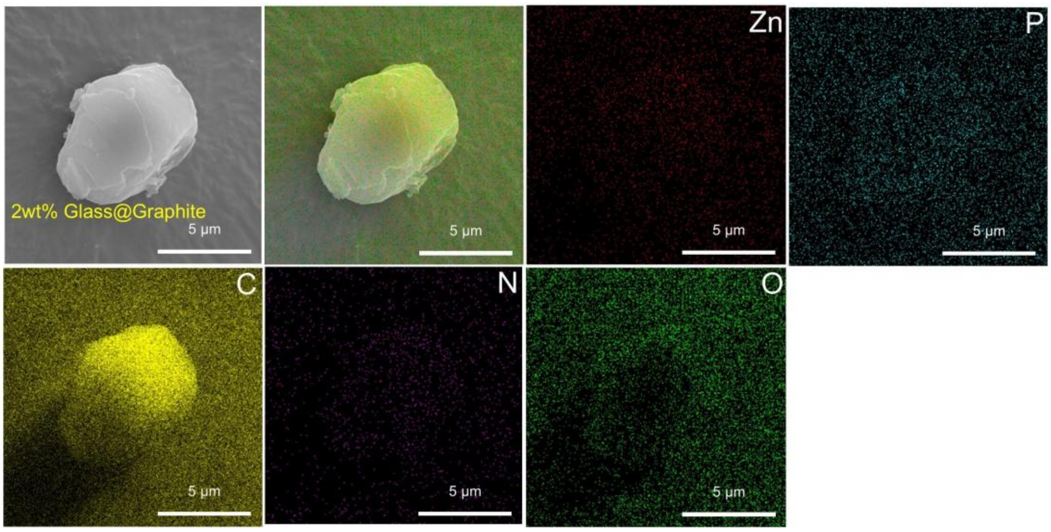

Figure S15. (a-d) SEM and (e-h) TEM images of 5 wt% Glass@Graphite.

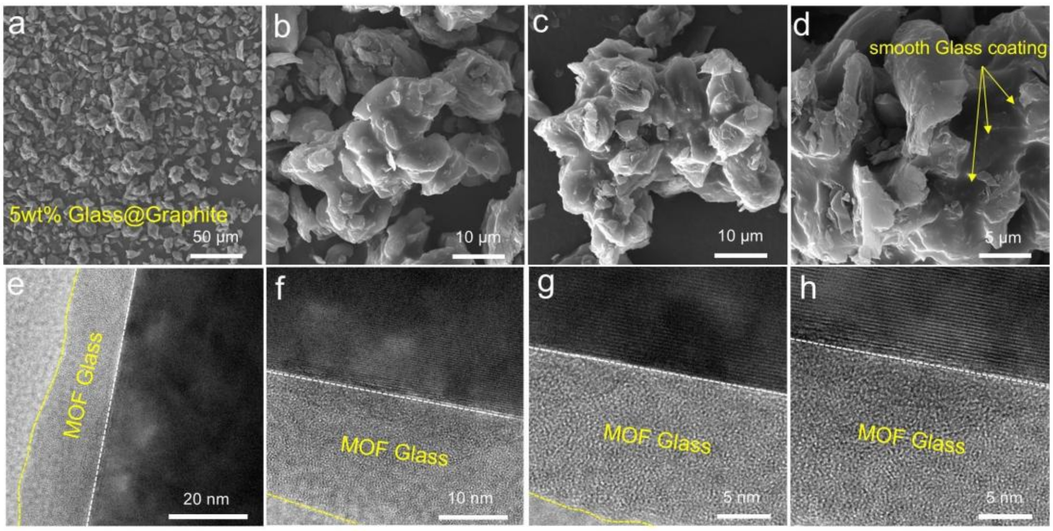

Figure S16. EDS elemental mapping images of 5 wt% Glass@Graphite.

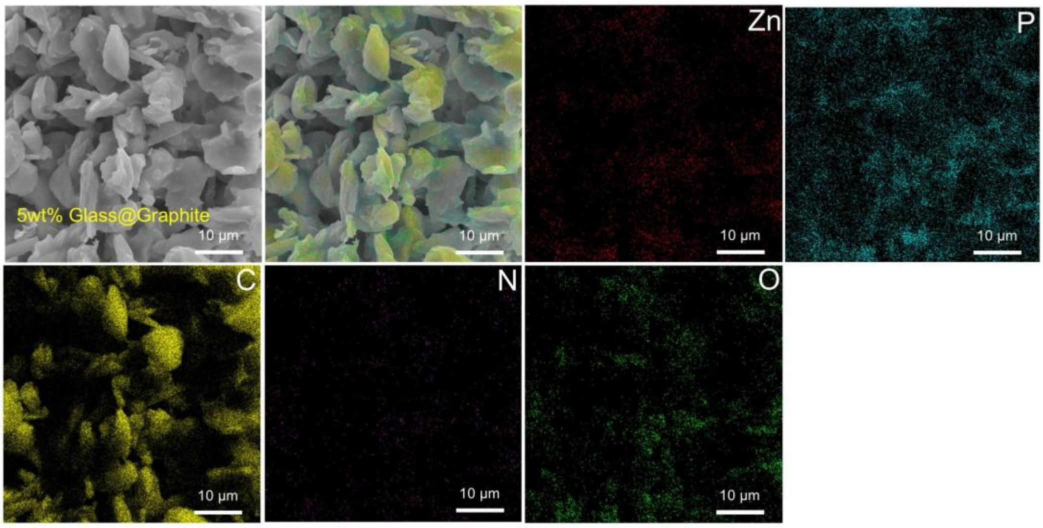

Figure S17. (a-d) SEM and (e-h) TEM images of 10 wt% Glass@Graphite.

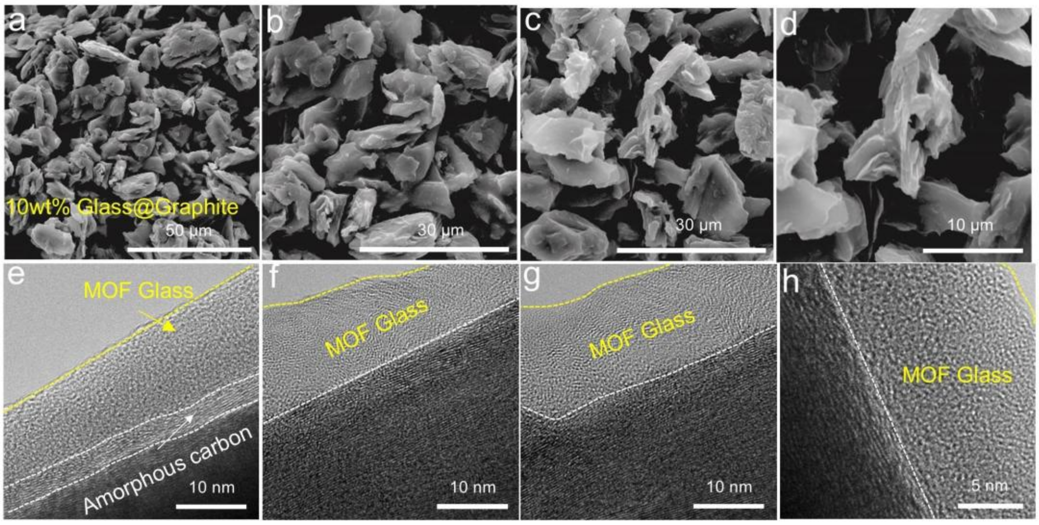

Figure S18. EDS elemental mapping images of 10 wt% Glass@Graphite.

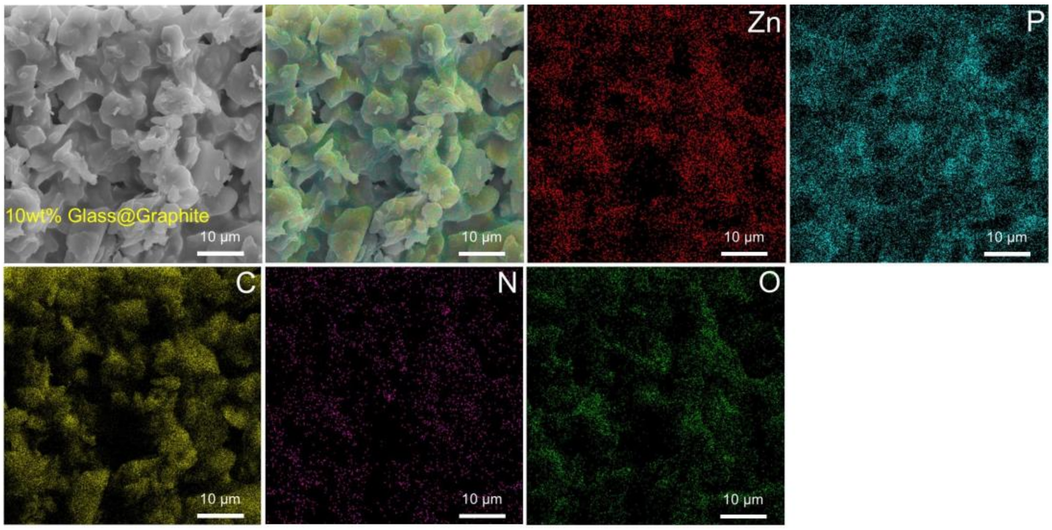

Figure S19. In-depth etching XPS of the cycled Glass@Graphite. (a) The overall in-depth etching XPS spectra of the cycled Glass@Graphite and the corresponding P 2p XPS spectra of the cycled Glass@Graphite (b) 4-times etching and (c) 0-time etching.

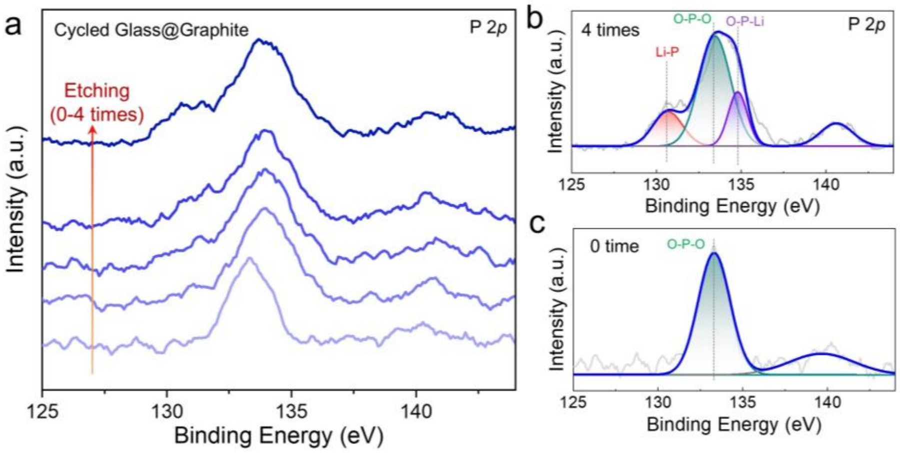

Figure S20. Contact angles of the electrolyte on Graphite electrode.

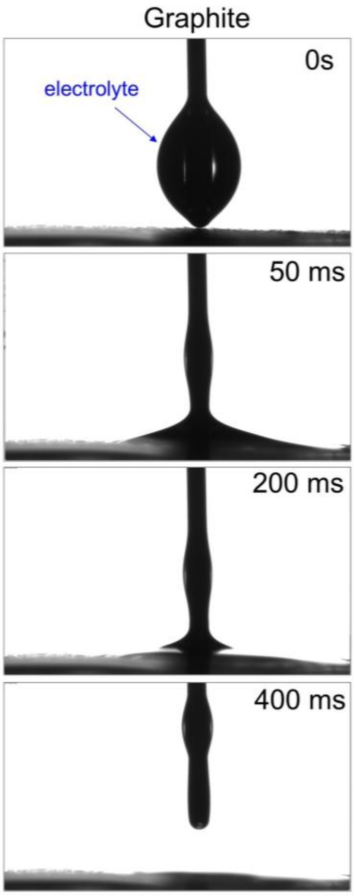

Figure S21. Cycling performance of half-cells assembled by Graphite and Glass@Graphite anode under different current densities with (a) 0.3 C and (b) 0.5 C.

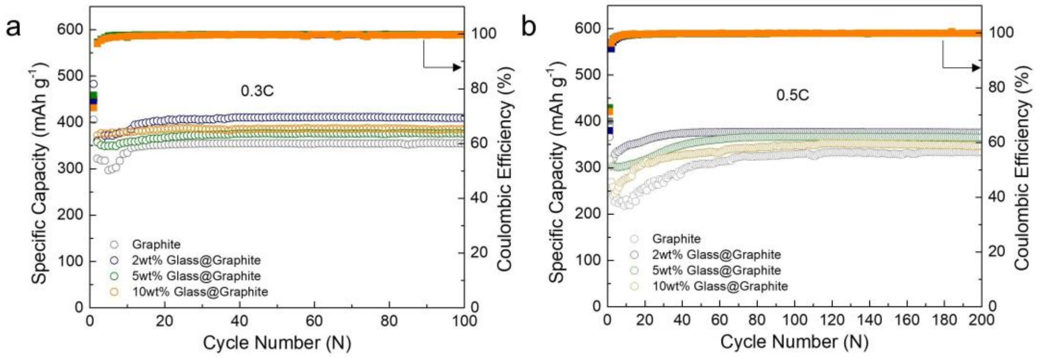

Figure S22. Galvanostatic charge-discharge voltage profiles for half-cells under different cycling numbers at the current density of 0.1 C. (a) Graphite, (b) 2 wt% Glass@Graphite, (c) 5 wt% Glass@Graphite, and (d) 10 wt% Glass@Graphite.

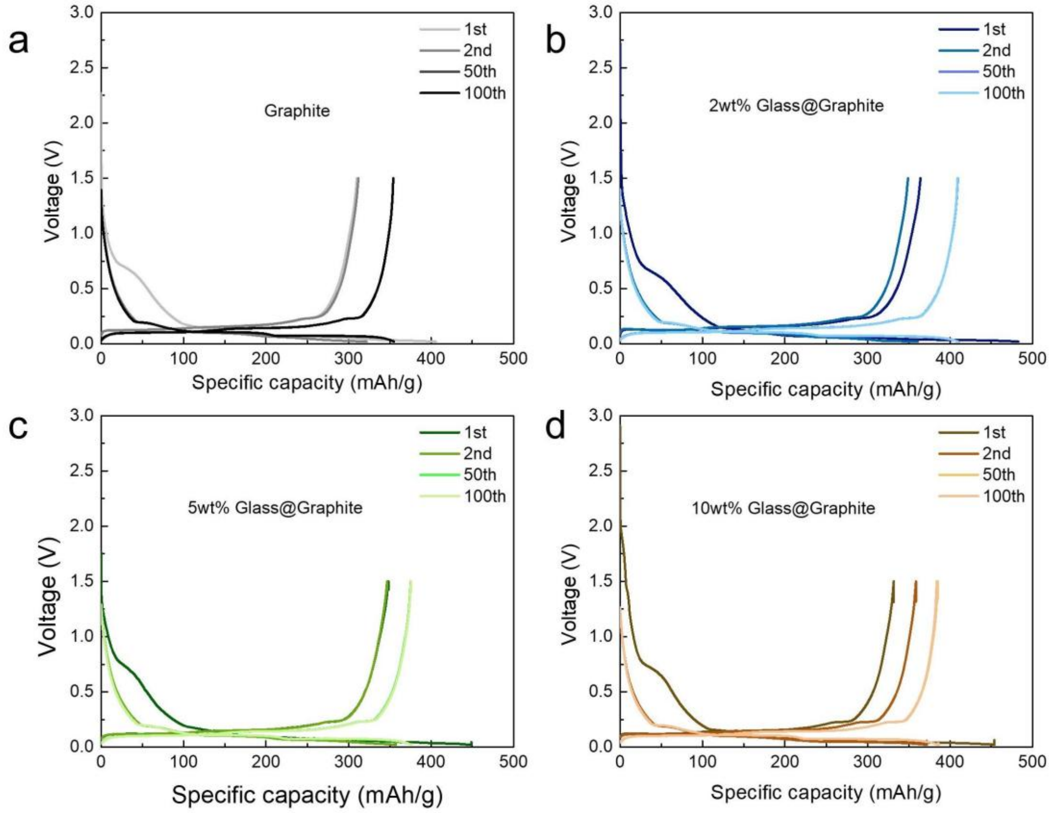

Figure S23. CV curves for half-cells based on (a) Graphite and (b-d) Glass@Graphite with different Glass coating proportions.

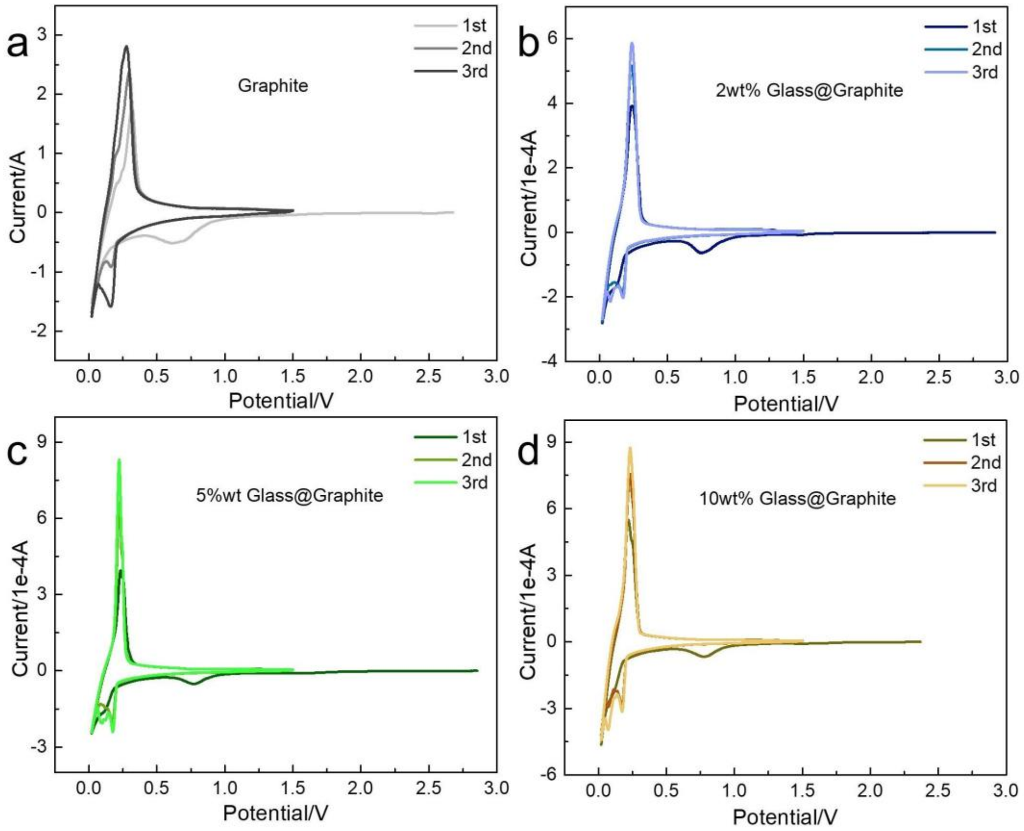

Figure S24. SEM images of Graphite anode after 200 cycles.

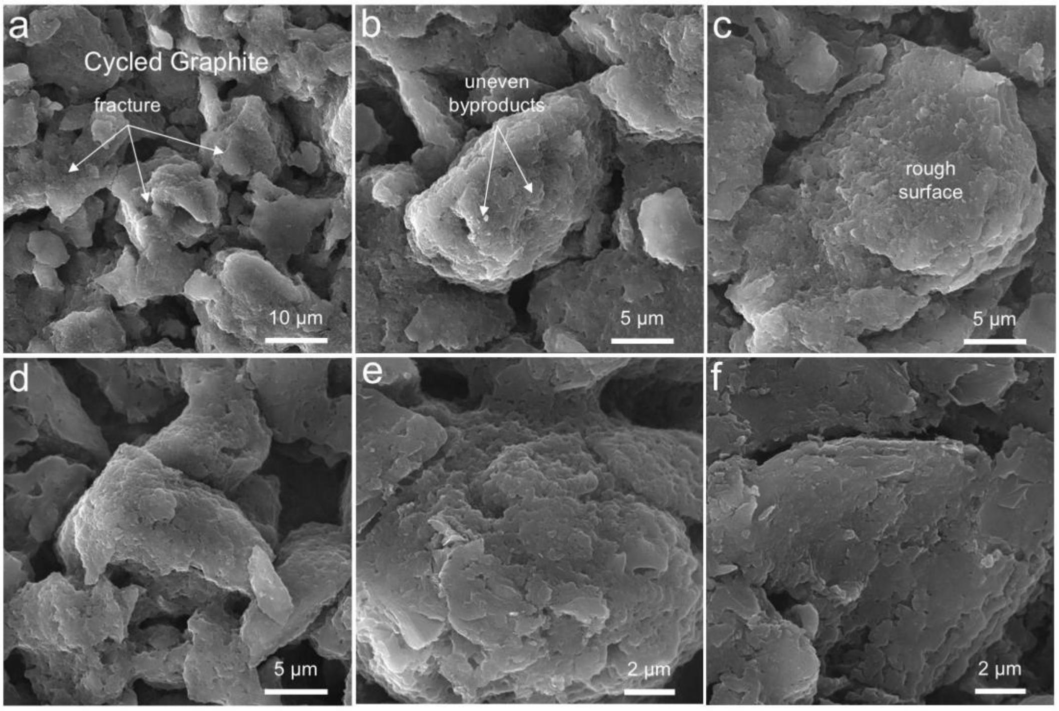

Figure S25. SEM images of 2 wt% Glass@Graphite anode after (a-c) 200 cycles and (d-f) 1000 cycles.

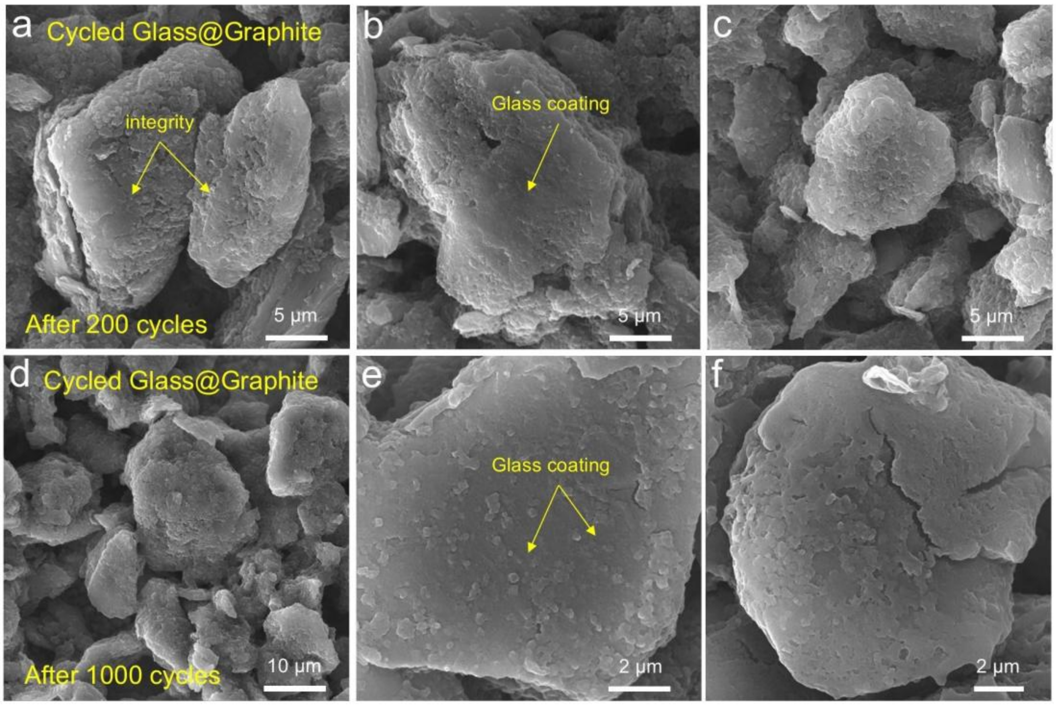

Figure S26. HRTEM images of Graphite anode after 200 cycles.

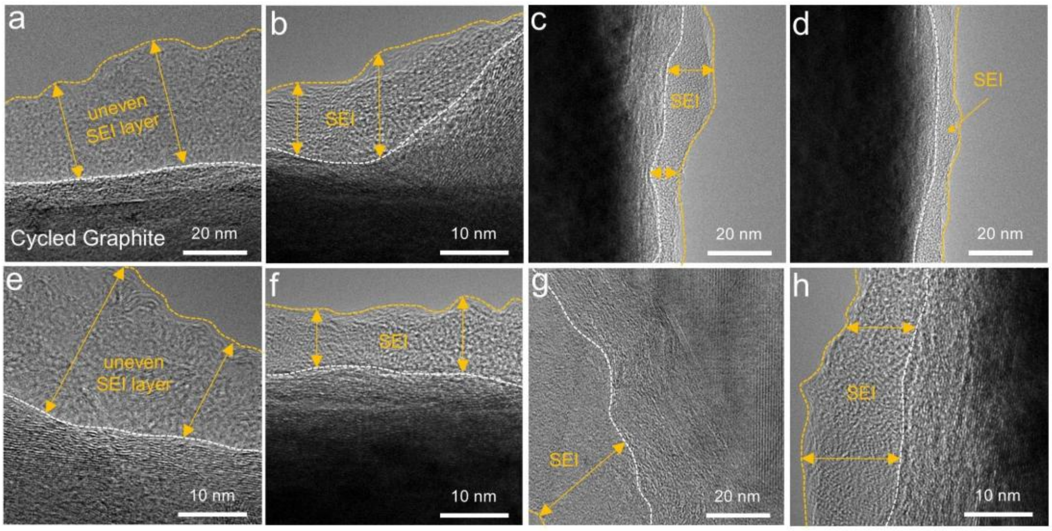

Figure S27. HRTEM images of 2 wt% Glass@Graphite anodes after 1000 cycles.

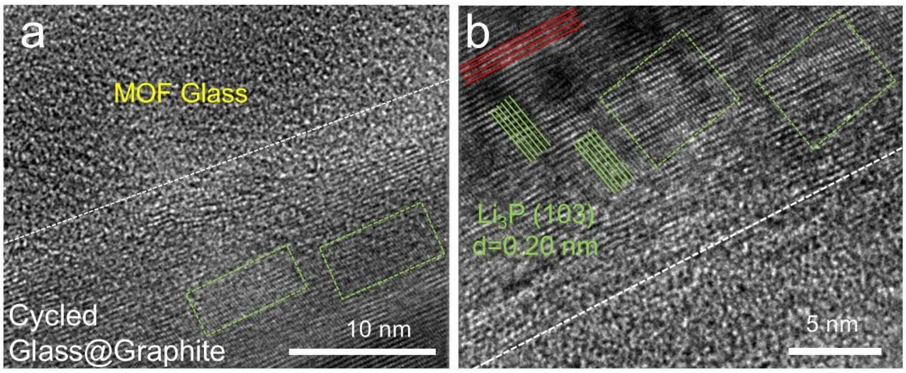

Figure S28. 2D views of elements distribution in the time-of-flight secondary-ion mass spectrometry (TOF-SIMS) sputtered volumes of (g) cycled Graphite and (h) cycled Glass@Graphite.

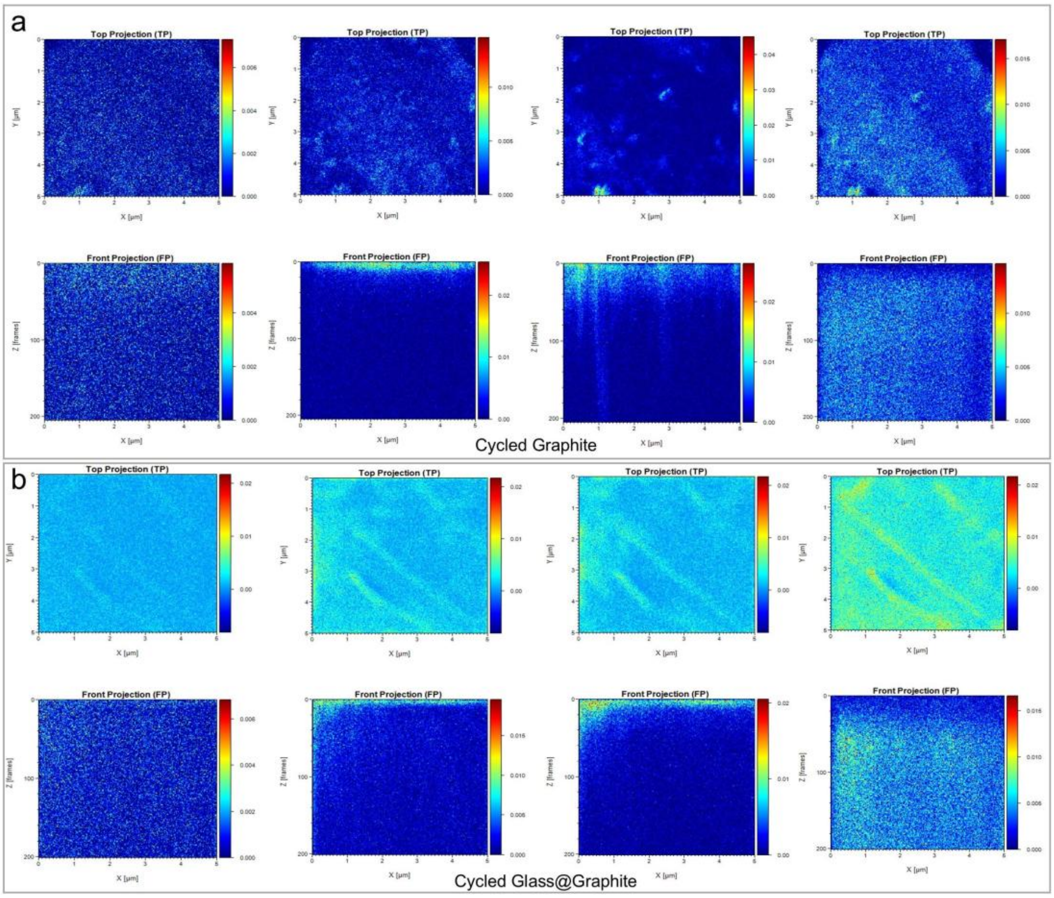

Figure S29. Depth-resolution etching FT-IR spectrum of (a) Graphite and (b) 2 wt% Glass@Graphite anodes after 200 cycles.

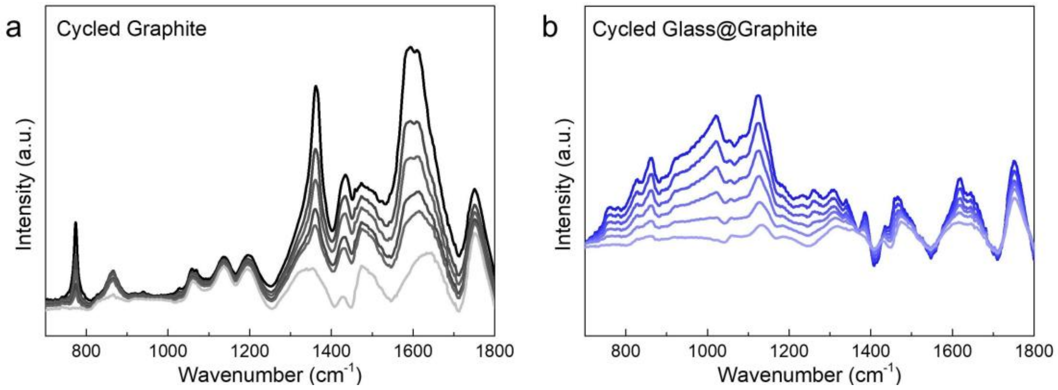

Figure S30. EIS curves of half-cells based on Graphite and 2 wt% Glass@Graphite anodes before and after cycling.

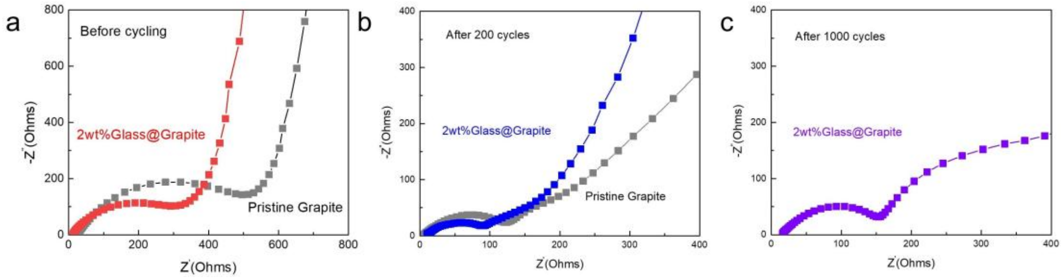

Supplemental References

(1) Li J, Wang J, Li Q, Zhang M, Li J, Sun C, Yuan S, Feng X, Wang B. Coordination polymer glasses with lava and healing ability for high-performance gas sieving[J]. Angew. Chem. Int. Ed, **2021**, 60(39): 21304-21309.

(2) Quan Z, Lu A, Wang F, Liu Z, Wang S, Zhou Y, Zhang C, Ye C, Liu J, Tan J. Soft carbon filled in expanded graphite layer pores for superior fast-charging lithium-ion batteries[J]. Carbon, **2024**, 229: 119500.

(3) Rhee D Y, Kim J, Moon J, Park M-S. Off-stoichiometric TiO_2-decorated graphite anode for high-power lithium-ion batteries[J]. J. Alloy. Compd., **2020**, 843(30): 156042.

(4) Cai W, Yan C, Yao Y X, Xu L, Xu R, Jiang L L, Huang J Q, Zhang Q. Rapid lithium diffusion in Order@Disorder pathways for fast-charging graphite anodes[J]. Small Struct., **2020**, 1(1): 2000010.

(5) Mu Y, Han M, Li J, Liang J, Yu J. Growing vertical graphene sheets on natural graphite for fast charging lithium-ion batteries[J]. Carbon, **2021**, 173: 477-484.

(6) Han Y J, Kim J, Yeo J S, An J C, Hong I-P, Nakabayashi K, Miyawaki J, Jung J D, Yoon S H. Coating of graphite anode with coal tar pitch as an effective precursor for enhancing the rate performance in Li-ion batteries: Effects of composition and softening points of coal tar pitch[J]. Carbon, **2015**, 94: 432-438.

(7) Shim J H, Lee S. Characterization of graphite etched with potassium hydroxide and its application in fast-rechargeable lithium-ion batteries[J]. J. Power Sources, **2016**, 324: 475-483.

(8) Zhou J, Ma K, Lian X, Shi Q, Wang J, Chen Z, Guo L, Liu Y, Bachmatiuk A, Sun J, Yang R, Choi J H, Rümmeli M H. Eliminating Graphite exfoliation with an artificial solid electrolyte interphase for stable lithium-ion batteries[J]. Small, **2022**, 18(15): 2107460.

(9) Chang Q, Li L, Zuo Z, Li Y. sp-carbon-enabled interface for high-performance graphite anode[J]. Nano Today, **2022**, 44.

(10) Deng L, Cai C, Huang Y, Fu Y. In-situ MOFs coating on 3D–channeled separator with superior electrolyte uptake capacity for ultrahigh cycle stability and dendrite-inhibited lithium-ion batteries[J]. Microporous Mesoporous Mater, 2022, 329: 111544.

(11) Liu N, Liu J, Jia D, Huang Y, Luo J, Mamat X, Yu Y, Dong Y, Hu G. Multi-core yolk-shell like mesoporous double carbon-coated silicon nanoparticles as anode materials for lithium-ion batteries[J]. Energy Storage Mater., 2019, 18: 165-173.

(12) Wang K, Pei S, He Z, Huang L-a, Zhu S, Guo J, Shao H, Wang J. Synthesis of a novel porous silicon microsphere@carbon core–shell composite via in situ MOF coating for lithium-ion battery anodes[J]. Chem. Eng. J, 2019, 356: 272-281.

(13) Yao L, Liang F, Jin J, Chowdari B V R, Yang J, Wen Z. Improved electrochemical property of Ni-rich LiNi0.6Co0.2Mn0.2O2 cathode via in-situ ZrO2 coating for high energy density lithium-ion batteries[J]. Chem. Eng. J, 2020, 389: 124403.

(14) Ding J, Du T, Jensen L R, Sørensen S S, Wang D, Wang S, Zhang L, Yue Y, Smedskjaer M M. High–performance dendrite–free Lithium metal anode based on metal–organic framework Glass[J]. Adv. Mater, 2024, 36(29): 2400652.
